# Supplementary material for: Complexation-driven assembly of imine-linked helical receptors showing adaptive folding and temperature-dependent guest selection
Source: Nat Commun. 2024 Feb 19;15:1501. doi: 10.1038/s41467-024-45322-y (PMC10876968; doi:10.1038/s41467-024-45322-y)
Supplement: Supplementary file 1 — Supplementary Information [file 41467_2024_45322_MOESM1_ESM.pdf]

Complexation-driven assembly of imine-linked helical receptors showing adaptive folding and temperature-dependent guest selection

Supplementary Information

Geunmoo Song[1], Seungwon Lee[1] and Kyu-Sung Jeong[1]*

[1] Department of Chemistry, Yonsei University, Seoul, 03722, Korea

E-mail: ksjeong@yonsei.ac.kr; Fax:+82-2-364-7050; Tel:+82-2-2123-2643

Contents

1. **Syntheses and characterization of new compounds**
2. **^{1}H NMR spectra and HPLC analyses of reaction mixtures**
   - 2.1 ^{1}H NMR spectra
   - 2.2 HPLC analyses
3. **^{1}H NMR studies**
   - 3.1 1D- and 2D-^{1}H NMR spectra and peak assignments for **4** and **4**⊃α-D-GF·H_2O
   - 3.2 ^{1}H NMR spectrum of **6** with the peak assignment
   - 3.3 ^{1}H NMR and ^{1}H-^{1}H ROESY spectra of (**6**-*MP*)$_2$⊃(me-β-D-glc)$_2$
   - 3.4 ^{1}H NMR and ^{1}H-^{1}H ROESY spectra of (**6**-*MM*)$_2$⊃(me-β-D-gal·2H_2O)$_2$
   - 3.5 Time-dependent ^{1}H NMR spectra for a 1:1 mixture of **4** and D-galactose
   - 3.6 ^{1}H NMR spectra of **4** and **6** in the presence of mixed guests
   - 3.7 Temperature-dependent ^{1}H NMR spectra for a mixture of **6**, me-β-D-glc, and me-β-D-gal
4. **CD spectra of 4**
5. **Binding studies**
   - 5.1 CD titrations of **4** with various guests
   - 5.2 ^{1}H NMR titrations of **6** with me-β-D-glc and me-β-D-gal
   - 5.3 CD titrations of **6** with me-β-D-gal
   - 5.4 UV-visible titrations of **6** with me-β-D-glc and me-β-D-gal
   - 5.5 Isothermal titration calorimetry (ITC) experiments of **6** with various guests
6. **X-ray crystallography analyses**
   - 6.1 Crystal growing
   - 6.2 Summary
7. **^{1}H, ^{13}C NMR, and mass spectra of new compounds**
8. **Supplementary references**

General: All chemicals were purchased from commercial suppliers and used without further purification unless otherwise specified. Dichloromethane (CH_2Cl_2) were purified by drying over calcium hydride (CaH_2), followed by distillation. Ethyl acetate (EtOAc) and acetone were distilled. A solvent mixture of 5% (or 10%) (v/v) (deuterated) DMSO/CH_2Cl_2 with water content ranging from 0.04% to 0.06% was prepared by combining dried DMSO with a 10:1 (v/v) mixture of anhydrous and water-saturated CH_2Cl_2. Organic solvents were dried over molecular sieves (4 Å). Water-saturated CH_2Cl_2 was prepared by sonicating the solvent with a few drops of distilled water for 10 min, and organic layer was carefully separated out for use. Water percentage was calculated based on ^{1}H NMR integration. Thin layer chromatography (TLC) was performed on Merck (silica gel 60, F-254, 0.25 mm). Silica gel 60 (230-400 mesh, Merck) was used for column chromatography. Melting points were determined with a Barnstead Electrothermal (IA9100) apparatus. NMR spectra were measured by using Bruker DRX 400, Avance II instruments and Avance III HD 300 instruments. Abbreviations used when describing NMR spectra are as follows: s, singlet; d, doublet; dd, doublet of doublet; t, triplet; m, multiplet. Coupling constants (*J*) are given in Hz. Chemical shifts are reported using residual protonated solvent peaks (for ^{1}H NMR spectra, DMSO-d_6 2.50 ppm; CD_2Cl_2 5.32 ppm; $(CD_2Cl)_2$ 3.72 ppm and for ^{13}C NMR spectra, DMSO-d_6 39.52 ppm; CD_2Cl_2 53.84 ppm). NMR data processing was achieved with Topspin 4.1.1 software. Complete assignments of receptor **4** and **6** were achieved by using a combination of 2D ^{1}H, ^{1}H-COSY and 2D ^{1}H, ^{1}H-ROESY (mixing time: 400 ms). Assignments of **4**⊃α-D-GF·H_2O, (**6**-*MP*)$_2$⊃(me-β-D-glc)$_2$, and (**6**-*MM*)$_2$⊃(me-β-D-gal·2H_2O)$_2$ complexes were based on 2D ^{1}H, ^{1}H-COSY, 2D ^{1}H, ^{1}H-ROESY (mixing time: 100 and 400 ms), and 2D ^{1}H, ^{13}C-HSQC. MALDI-TOF/TOF mass spectrometric measurement was performed on Bruker (Autoflex Max). The ESI-HRMS spectrometric measurements were obtained from the *Organic Chemistry Research Center* at Sogang University. Q-EXACTIVE PLUS ORBITRAP MS (Thermo scientific) spectrometric measurement was obtained at from *Research Facilities Center* at Yonsei University.

1. Syntheses and characterization of new compounds

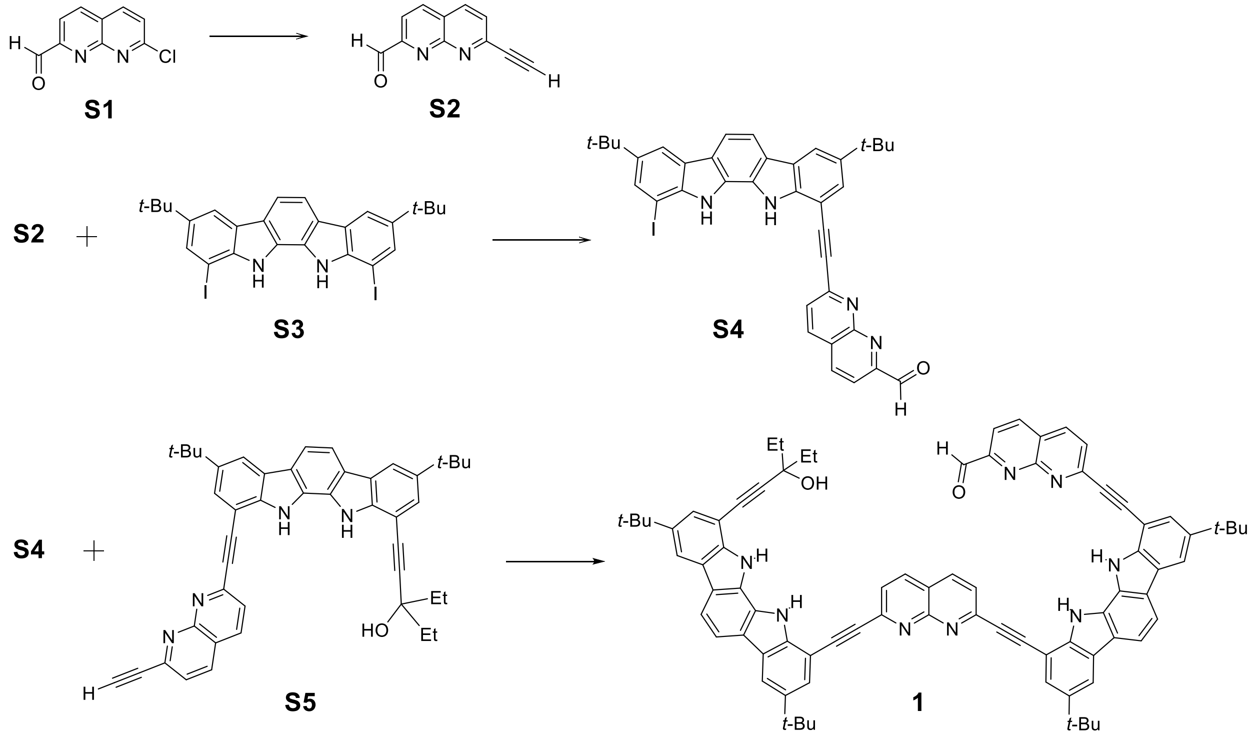

S2: A Schlenk flask containing **S1**[1] (540 mg, 2.8 mmol), CuI (10.6 mg, 0.056 mmol) and $Pd(PPh_3)_2Cl_2$ (39 mg, 0.056 mmol) was evacuated under vacuum and back-filled with nitrogen. Anhydrous, degassed tetrahydrofuran (THF) (7.0 mL), triethylamine (Et_3N) (1.6 mL), and trimethylsilylacetylene (0.6 mL, 4.2 mmol) were sequentially added and the solution was stirred at 55 °C for 3.5 h. The reaction mixture was cooled to room temperature, filtered through Celite with CH_2Cl_2 and concentrated. The residue was dissolved in CH_2Cl_2, washed with brine, and dried over anhydrous Na_2SO_4. The residue was then dissolved in THF (10 mL) and methanol (2 mL), and solid K_2CO_3 (387 mg) was added. After 20 min stirring at room temperature, the residue was concentrated and purified by flash column chromatography (silica gel, hexanes : CH_2Cl_2 : ethyl acetate (EtOAc) = 1:2:2 (v/v/v)) to give compound **S2** (265 mg, 53%) as a grey solid; mp > 165 °C (dec); TLC (hexanes : tetrahydrofuran (THF)) = 1:1 v/v) Rf = 0.35 ; ^{1}H NMR (400 MHz, DMSO-d_6, 25 °C, ppm) δ 10.14 (s, 1H), 8.72 (d, J = 8.3, 1H), 8.64 (d, J = 8.4, 1H), 8.08 (d, J = 8.3, 1H), 7.89 (d, J = 8.4, 1H), 4.77 (s, 1H); ^{13}C NMR (100 MHz, DMSO-d_6, 25 °C, ppm) δ 193.7, 155.2, 154.7, 146.5, 139.8, 138.8, 127.1, 124.6, 118.6, 83.6, 82.9; ESI-HRMS, m/z calcd for $C_{11}H_6N_2O$ $[M+Na]^+$ 205.0372 found 205.0372

S4: A Schlenk flask containing **S2** (201 mg, 1.1 mmol), **S3**[2] (684 mg, 1.1 mmol), CuI (6.3 mg, 0.033 mmol), and $Pd(PPh_3)_2Cl_2$ (23 mg, 0.033 mmol) was evacuated under vacuum and back-filled with

nitrogen. Anhydrous, degassed dimethylformamide (DMF) (11 mL) and triethylamine (Et_3N) (0.6 mL) were sequentially added, and the solution was stirred at 45 °C for 2 h. The mixture was cooled to room temperature, filtered through Celite with CH_2Cl_2, and concentrated. The residue was dissolved in CH_2Cl_2, washed with brine, and dried over anhydrous Na_2SO_4. After concentrated, the residue was purified by flash column chromatography (silica gel, hexanes : CH_2Cl_2 : ethyl acetate (EtOAc) = 1:1:0.2 (v/v/v)) to give compound **S4** (322 mg, 43%) as a yellow solid; mp > 225 °C (dec); TLC (hexanes : tetrahydrofuran (THF) = 1:1 v/v) Rf = 0.45; ^{1}H NMR (400 MHz, 10% (v/v) DMSO-d_6/CD_2Cl_2, 25 °C, ppm) δ 11.25 (s, 1H, NH), 10.83 (s, 1H, NH), 10.15 (s, 1H), 8.32 (d, J = 8.4, 1H), 8.24 (d, J = 8.4, 1H), 8.23 (s, 1H), 8.09 (s, 1H), 7.97 (d, J = 8.3, 1H), 7.90 (d, J = 8.3, 1H), 7.87 (d, J = 8.3, 1H), 7.85 (d, J = 8.3, 1H), 7.80 (s, 1H), 7.80 (s, 1H), 1.49 (s, 9H, *t*-Bu), 1.43 (s, 9H, *t*-Bu); ^{13}C NMR (100 MHz, 10% (v/v) DMSO-d_6/CD_2Cl_2, 25 °C, ppm) δ 193.6, 155.9, 155.5, 148.4, 144.5, 142.6, 139.7, 138.9, 138.9, 138.8, 137.6, 131.5, 127.6, 127.2, 126.7, 126.3, 124.6, 124.5, 121.8, 121.0, 119.2, 118.2, 116.4, 112.5, 112.2, 103.4, 92.9, 90.9, 76.0, 34.9, 34.8, 32.0, 32.0; MALDI-TOF/TOF, m/z calcd for $C_{37}H_{31}IN_4O$ $[M+H]^+$ 675.2 found 675.3

1: A Schlenk flask containing **S4** (417 mg, 0.62 mmol), **S5**[3] (466 mg, 0.71 mmol), CuI (5.9 mg, 0.031 mmol) and $Pd(PPh_3)_2Cl_2$ (22 mg, 0.031 mmol) was evacuated under vacuum and back-filled with nitrogen. Anhydrous, degassed dimethylformamide (DMF) (6.3 mL) and triethylamine (Et_3N) (0.35 mL) were sequentially added, and the solution was stirred at 45 °C for 3.5 h. The mixture was cooled to room temperature, filtered through Celite with CH_2Cl_2, and concentrated. The residue was dissolved in CH_2Cl_2, washed with brine, and dried over anhydrous Na_2SO_4. After concentrated, the residue was purified by flash column chromatography (silica gel, hexanes : tetrahydrofuran (THF) = 1:1 (v/v)), and washed with hexanes, acetone, and ethyl acetate (EtOAc) to give compound **1** (460 mg, 61%) as an orange solid; mp > 264 °C (dec); TLC (hexanes : tetrahydrofuran (THF) = 1:1 v/v) Rf = 0.5; ^{1}H NMR (400 MHz, DMSO-d_6, 25 °C, ppm) δ 11.64 (s, 1H, NH), 11.54 (s, 1H, NH), 11.44 (s, 1H, NH), 10.76 (s, 1H, NH), 9.95 (s, 1H), 8.71 (s, 1H), 8.69 (s, 1H), 8.45 (s, 2H), 8.38(d, J = 1.5, 1H), 8.31 (d, J = 8.4, 1H), 8.23 (s, 1H), 8.22 (d, J = 8.0, 1H), 8.13 (s, 3H), 8.11 (d, J = 1.5, 1H), 8.05 (d, J = 8.4, 1H), 8.02 (d, J = 8.4, 1H), 7.91 (d, J = 8.3, 1H), 7.84 (d, J = 1.4, 1H), 7.81 (d, J = 1.4, 1H), 7.72 (d, J = 1.4, 1H), 7.59 (d, J = 8.3, 1H), 7.44 (d, J = 1.4, 1H), 5.18 (s, 1H, OH), 1.72–1.56 (m, 4H), 1.50 (s, 9H, *t*-Bu), 1.49 (s, 9H, *t*-Bu), 1.46 (s, 9H, *t*-Bu), 1.43 (s, 9H, *t*-Bu), 0.93 (*t*, J = 7.3, 6H); ^{13}C NMR (100 MHz, DMSO-d_6, 25 °C, ppm) δ 193.0, 155.5, 154.8, 154.7, 147.1, 146.8, 146.8, 142.2, 142.2, 142.1, 141.8, 139.0, 138.6, 138.4, 138.3, 138.1, 138.0, 137.9, 137.8, 137.1, 127.0, 126.8, 126.5, 126.4, 126.0, 126.0, 126.0, 125.9, 125.8, 125.7, 124.2, 124.2, 124.1, 124.0, 123.7, 121.6, 120.9, 120.7, 120.7, 120.4, 119.3, 119.2, 119.1, 117.6, 116.8, 112.6, 112.4, 112.2, 105.0, 103.0, 102.8, 102.7, 97.6, 93.2, 92.8, 89.6, 89.4, 88.9, 79.7, 70.9, 70.9, 34.6, 34.6, 34.5, 34.4, 33.8, 31.8, 31.7, 8.7; ESI-HRMS, m/z calcd for $C_{82}H_{72}N_8O_2$ $[M+H]^+$ 1201.5851 found 1201.5868

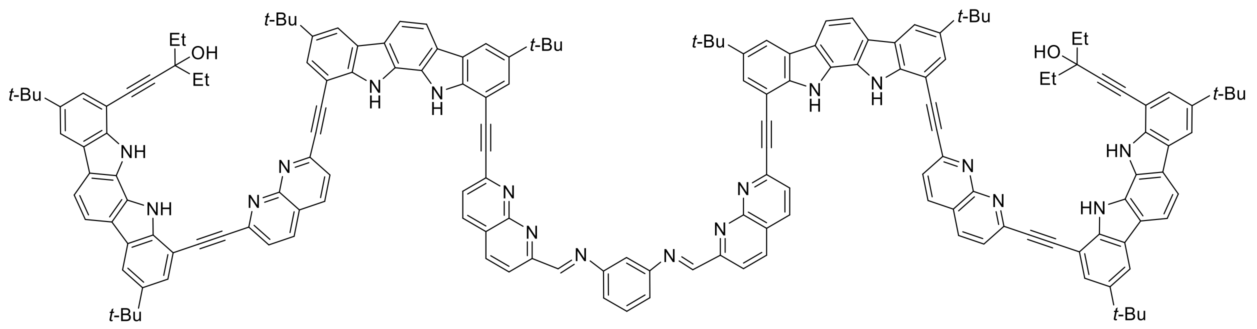

4

4: Synthesis of **4** is included in the methods section. mp > 254 °C (dec); TLC (hexanes : ethyl acetate (EtOAc) : methanol : triethylamine (TEA) = 5:5:1:1 v/v/v/v) Rf = 0.65; ^{1}H NMR (400 MHz, DMSO-d_6, 25 °C, ppm) δ 11.66 (s, 1H, NH), 11.54 (s, 1H, NH), 11.22 (s, 1H, NH), 10.66 (s, 1H, NH), 8.57 (s, 1H), 8.53 (d, *J* = 8.3, 1H), 8.43 (d, *J* = 8.3, 1H), 8.40 (s, 2H), 8.32 (s, 1H), 8.09 (s, 1H), 8.08 (s, 2H), 8.02 (d, *J* = 8.3, 1H), 8.01 (s, 1H), 7.99 (d, *J* = 8.3, 1H), 7.93 (d, *J* = 8.4, 1H), 7.83 (d, *J* = 8.4, 1H), 7.82 (d, *J* = 8.2, 1H), 7.78 (s, 1H), 7.77 (s, 1H), 7.72 (d, *J* = 8.3, 1H), 7.63 (d, *J* = 8.3, 1H), 7.54 (s, 1H), 7.49 (d, *J* = 8.0, 0.5H), 7.39 (s, 1H), 7.25 (d, *J* = 8.3, 1H), 7.03 (s, 0.5H), 5.11 (s, 1H, OH), 1.65–1.53 (m, 4H), 1.49 (s, 9H, *t*-Bu), 1.48 (s, 9H, *t*-Bu), 1.36 (s, 9H, *t*-Bu), 1.34 (s, 9H, *t*-Bu), 0.90 (t, *J* = 7.3, 6H); ^{13}C NMR (100 MHz, DMSO-d_6, 25 °C, ppm) *δ* 160.4, 157.1, 155.4, 154.9, 150.8, 146.7, 146.6, 146.1, 142.1, 142.1, 141.8, 141.6, 138.1, 138.0, 137.9, 137.9, 137.8, 137.6, 137.4, 137.1, 126.6, 126.4, 126.2, 126.0, 126.0, 125.9, 125.8, 125.8, 125.8, 125.6, 124.2, 124.2, 124.2, 123.6, 122.4, 121.4, 120.8, 120.7, 120.6, 120.4, 119.9, 119.2, 119.1, 118.6, 116.6, 112.5, 112.2, 112.1, 105.0, 103.0, 102.9, 102.6, 97.4, 93.2, 92.9, 92.8, 89.2, 88.9, 88.8, 79.7, 70.8, 70.8, 67.3, 67.2, 34.6, 34.4, 34.3, 33.8, 31.8, 31.8, 31.7, 31.6, 8.7; ESI-HRMS, m/z calcd for $C_{170}H_{148}N_{18}O_2$ $[M+2H]^{2+}$ 1237.6089; found 1237.6101.

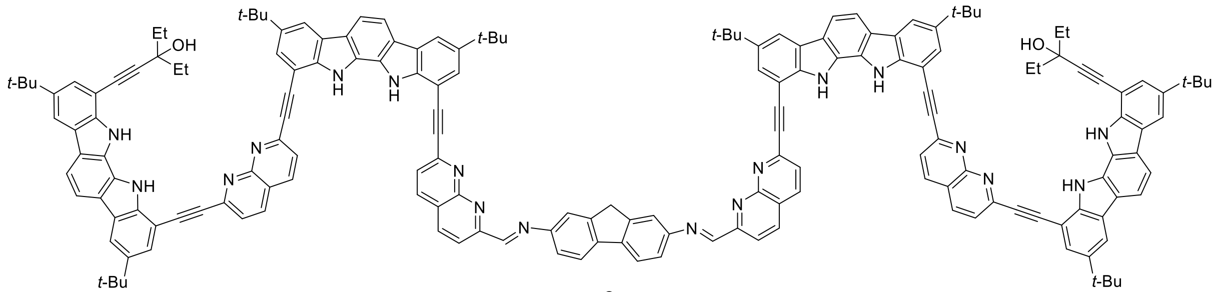

6

6: Synthesis of **6** is included in the methods section. mp > 242 °C (dec); TLC (hexanes : tetrahydrofuran (THF) : methanol : triethylamine (TEA) = 5:5:1:1 v/v/v/v) Rf = 0.7; ^{1}H NMR (400 MHz, DMSO-d_6, 25 °C, ppm) *δ* 11.66 (s, 1H, NH), 11.53 (s, 1H, NH), 11.37 (s, 1H, NH), 10.70 (s, 1H, NH), 8.69 (s, 1H), 8.65 (d, *J* = 8.2, 1H), 8.51 (d, *J* = 8.3, 1H), 8.45 (s, 2H), 8.36 (s, 1H), 8.23 (d, *J* = 8.4, 1H), 8.18 (s, 1H), 8.13 (s, 2H), 8.12 (d, *J* = 8.3, 1H), 8.05 (d, *J* = 8.5, 1H), 8.01 (d, *J* = 8.5, 1H), 7.97 (d, *J* = 8.5, 1H), 7.92 (s, 1H), 7.90 (s, 1H), 7.88 (d, *J* = 8.4, 1H), 7.85 (d, *J* = 8.4, 1H), 7.84 (s, 1H), 7.83.(s, 1H), 7.63 (s, 1H), 7.43 (d, *J* = 7.8,

2H), 7.29 (d, *J* = 8.4, 1H), 5.17 (s, 1H, OH), 3.78 (s, 1H), 1.71–1.59 (m, 4H), 1.50 (s, 9H, *t*-Bu), 1.50 (s, 9H, *t*-Bu), 1.41 (s, 9H, *t*-Bu), 1.37 (s, 9H, *t*-Bu), 0.95 (t, *J* = 7.3, 6H); ^{13}C NMR (100 MHz, DMSO-d_6, 25 °C, ppm) δ 158.7, 157.6, 155.4, 155.0, 148.4, 146.8, 146.7, 146.1, 144.5, 142.2, 142.1, 141.8, 141.7, 140.1, 138.2, 138.0, 138.0, 137.9, 137.8, 137.7, 137.4, 137.2, 126.4, 126.3, 126.0, 126.0, 125.9, 125.9, 125.9, 125.8, 125.8, 125.8, 125.7, 124.2, 124.2, 124.2, 123.7, 122.4, 121.3, 120.8, 120.7, 120.6, 120.5, 120.4, 119.2, 119.0, 118.7, 117.8, 116.7, 112.6, 112.6, 112.3, 112.1, 105.0, 103.1, 102.9, 102.7, 97.5, 93.2, 93.0, 92.9, 89.5, 89.0, 88.7, 79.7, 70.9, 70.9, 34.6, 34.5, 34.3, 33.8, 31.8, 31.8, 31.7, 31.7, 8.7; Q-Exactive Plus Orbitrap MS, m/z calcd for $C_{177}H_{152}N_{18}O_2$ $[M+2H]^{2+}$ 1281.6246; found 1281.6254.

2. ^{1}H NMR spectra and HPLC analyses of reaction mixtures

2.1 ^{1}H NMR spectra

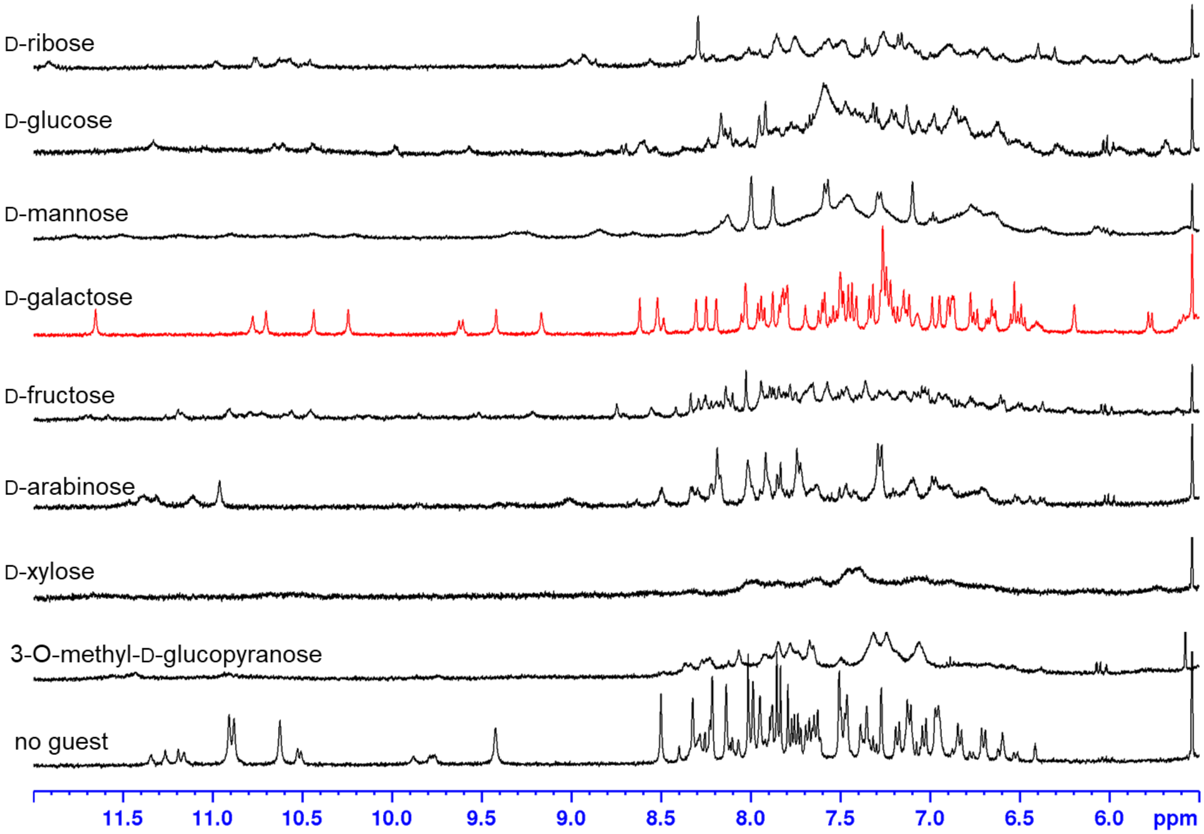

Supplementary Figure 1. Partial ^{1}H NMR spectra (400 MHz, 2% (v/v) DMSO-d_6/CD_2Cl_2, 25 °C) of reaction mixtures containing tetramer (**1**) (2 equiv.), benzene-1,3-diamine (**2**) (1 equiv.), chloroacetic acid (0.2 equiv.), and various guests (3 equiv.) after 24 h heating at 39 ± 1 °C.

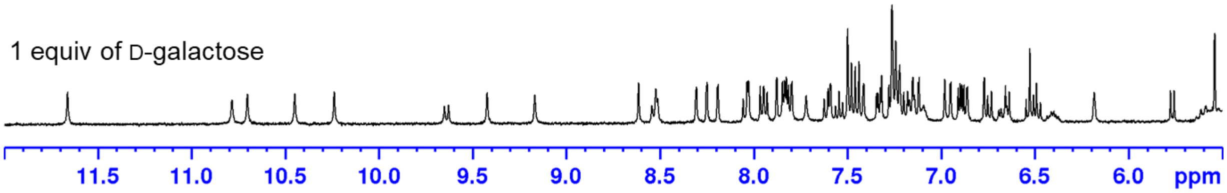

Supplementary Figure 2. Partial ^{1}H NMR spectrum (400 MHz, 5% (v/v) DMSO-d_6/CD_2Cl_2, 25 °C) of a reaction mixture containing tetramer (**1**) (2 equiv.), benzene-1,3-diamine (**2**) (1 equiv.), chloroacetic acid (0.2 equiv.), and D-galactose (1 equiv.) after 24 h heating at 39 ± 1 °C.

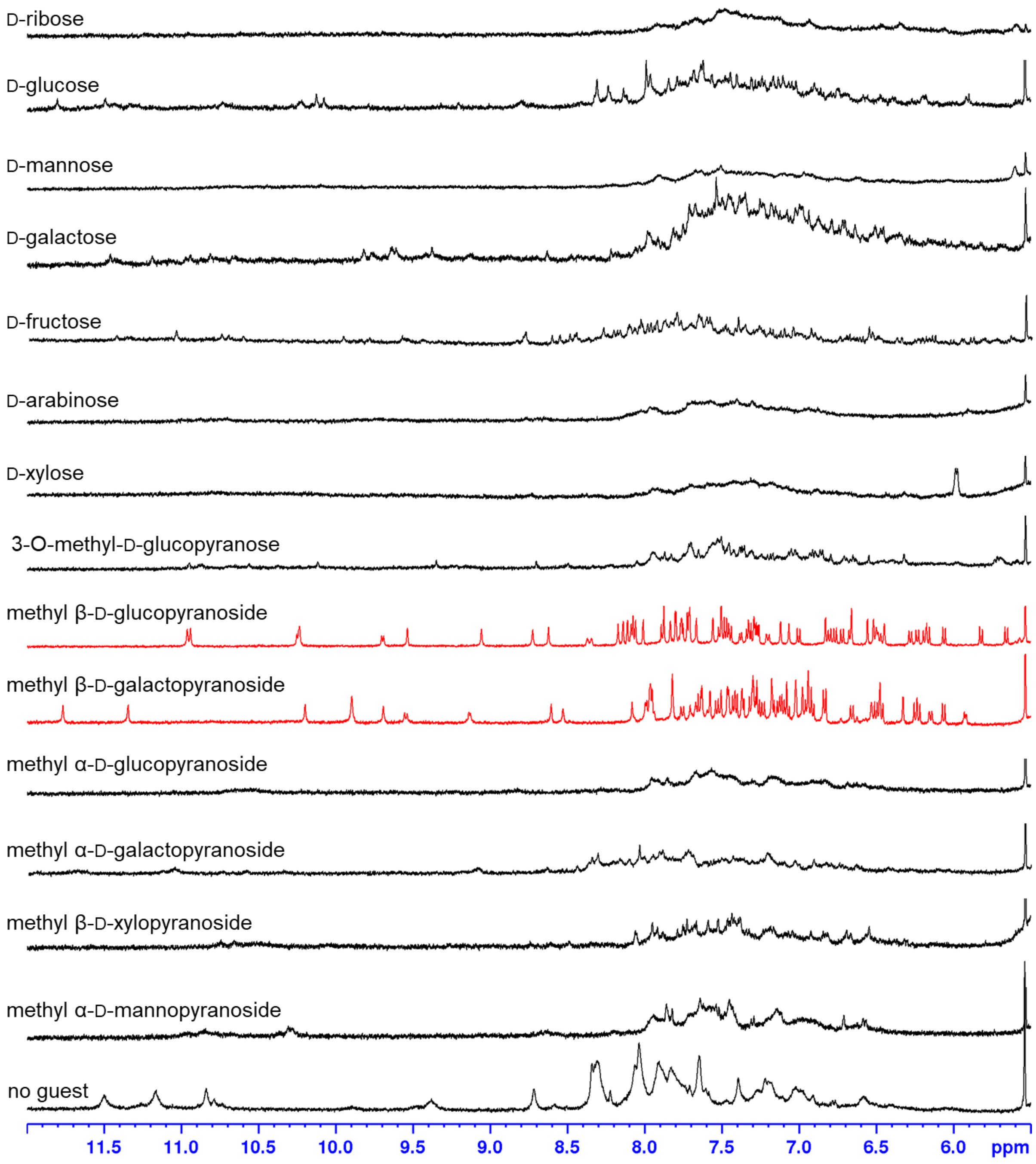

Supplementary Figure 3. Partial ^{1}H NMR spectra (400 MHz, 2% (v/v) DMSO-d_6/CD_2Cl_2, 25 °C) of reaction mixtures containing tetramer (**1**) (2 equiv.), 9*H*-fluorene-2,7-diamine (**5**) (1 equiv.), chloroacetic acid (0.2 equiv.), and various guests (3 equiv.) after 24 h heating at 39 ± 1 °C.

2.2 HPLC analyses

For HPLC analysis, reaction mixtures were filtered through a short column filled with basic silica. HPLC analyses were performed on JASCO HPLC. Samples were separated on a COSMOSIL 5SL-II Packed Column (4.6 mm I.D.× 250 mm). A flow rate of 0.2 mL min^{-1}, detection wavelength of 350 nm, column temperature of 25 °C, and column eluent of *n*-hexane : tetrahydrofuran (THF) : methanol : triethylamine (TEA) = 1:1:0.025:0.025 were applied. All solvents of HPLC grade were used. Quantitative anaylsis was conducted utilizing the peak areas corresponding to tetramer (**1**), monoimine (**3**), and diimine (**4**).

Supplementary Table 1. Estimated yields of tetramer (**1**), monoimine (**3**), and diimine (**4**) after imine reaction in various guests.

guest	Estimated yield (%)		
	**1**	**3**	**4**
D-ribose	20	18	62
D-glucose	11	8	81
D-mannose	5	4	91
D-galactose	-	-	100
D-fructose	19	15	66
D-arabinose	19	10	71
D-xylose	18	17	65
3-O-methyl-D-glucopyranose	29	21	50
no guest	29	28	43

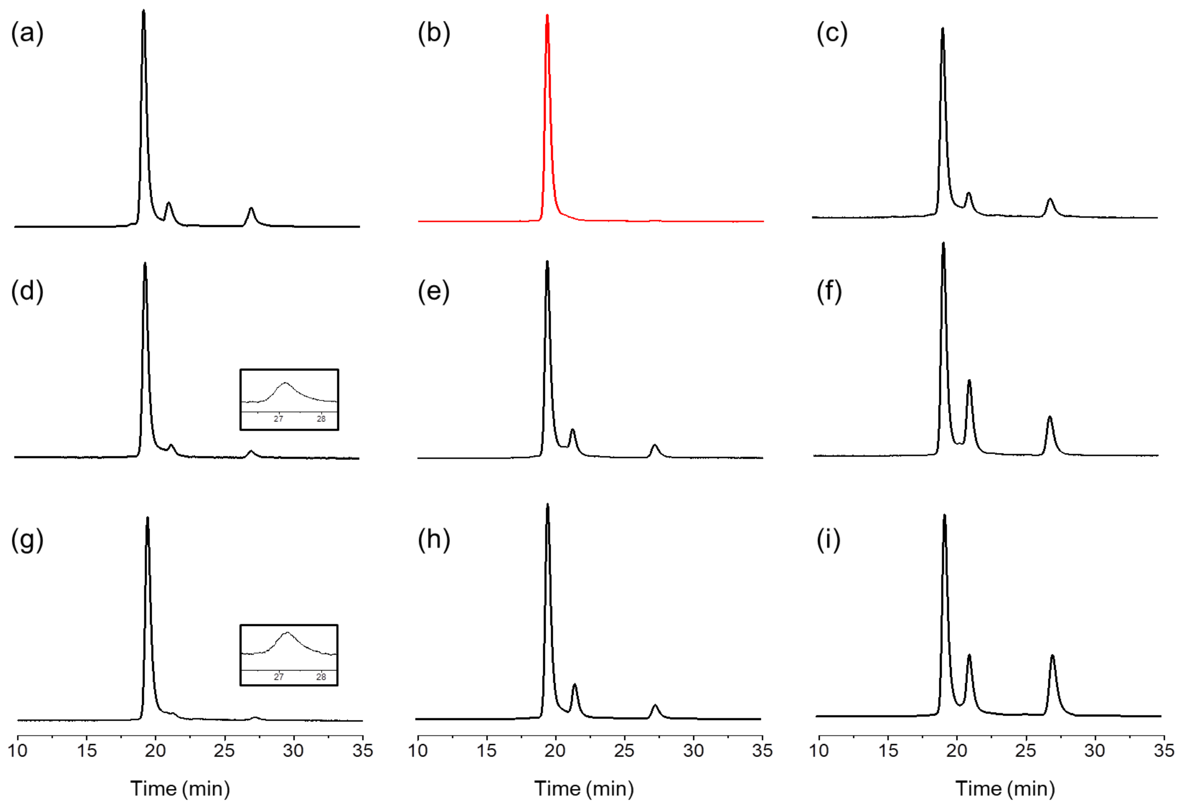

Supplementary Figure 4. HPLC traces of reaction mixtures obtained from tetramer (**1**) (2 equiv.), benzene-1,3-diamine (**2**) (1 equiv.), and chloroacetic acid (0.2 equiv.), in the presence of (a) D-ribose, (b) D-galactose, (c) D-xylose, (d) D-glucose, (e) D-fructose, (f) 3-O-methyl-D-glucopyranose, (g) D-mannose, (h) D-arabinose, (3 equiv. each) or (i) in the absence of guest after 24 h heating at 39 ± 1 °C. The retention times are 19.4 min for diimine (**4**), 21.3 min for tetramer (**1**), and 27.1 min for monoimine (**3**).

3. ^{1}H NMR studies

3.1 1D- and 2D-^{1}H NMR spectra and peak assignments for **4** and **4**⊃α-D-GF·H_2O

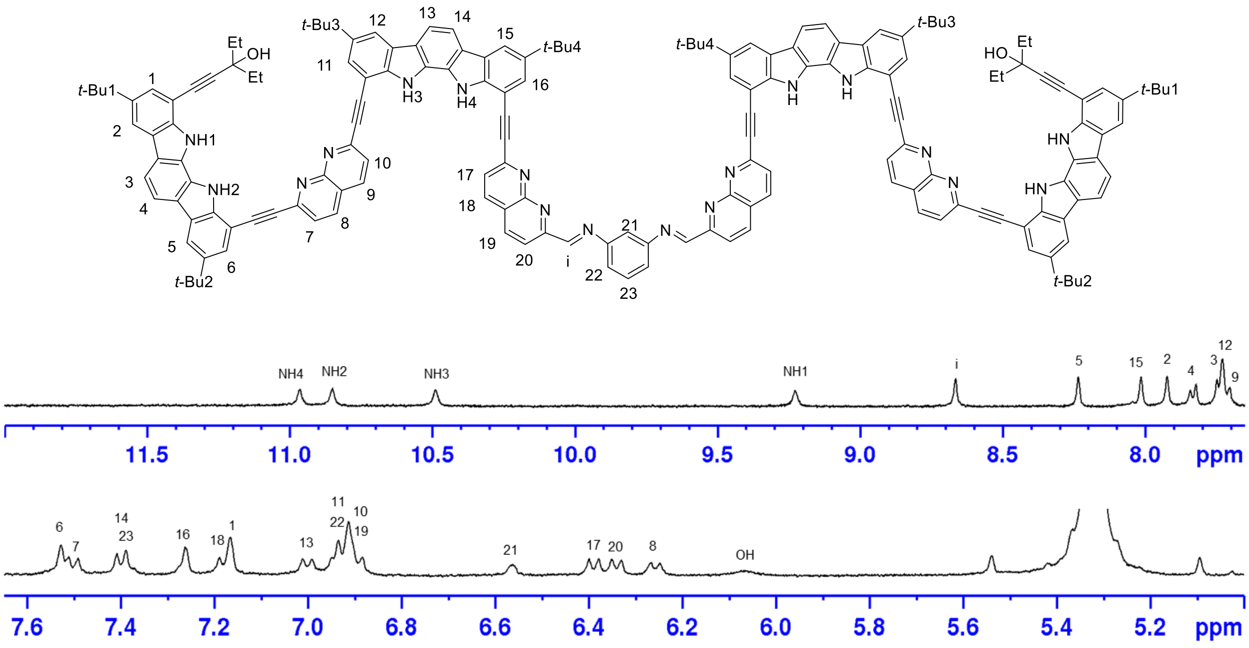

Supplementary Figure 5. Partial ^{1}H NMR spectrum (400 MHz, 25 °C) of **4** in CD_2Cl_2.

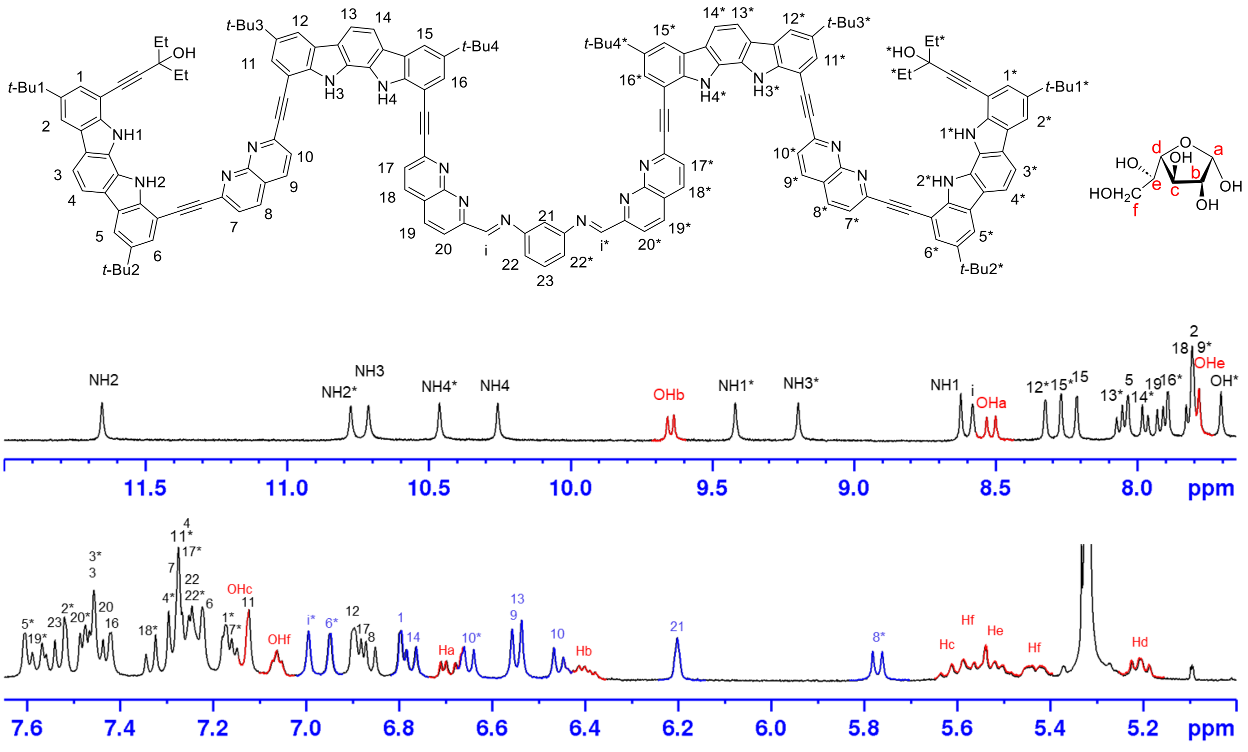

Supplementary Figure 6. Partial ^{1}H NMR spectrum (400 MHz, 25 °C) of **4**⊃α-D-GF·H_2O in CD_2Cl_2. Upfield shifted aromatic CH signals are depicted in blue, and α-D-GF signals are in red.

Supplementary Table 2. ^1H NMR chemical shifts (400 MHz, 25 °C) of **4** and **4**⊃α-D-GF·H_2O in CD_2Cl_2.

**4**		**4**⊃α-D-GF·H_2O			
Atom	^1H (ppm)	Atom	^1H (ppm)	Atom	^1H (ppm)
NH1	9.22 (s)	NH1	8.62 (s)	NH1*	9.42 (s)
NH2	10.85 (s)	NH2	11.65 (s)	NH2*	10.78 (s)
NH3	10.49 (s)	NH3	10.71 (s)	NH3*	9.20 (s)
NH4	10.96 (s)	NH4	10.26 (s)	NH4*	10.46 (s)
1	7.17 (s)	1	6.80 (d, *J* = 1.4 Hz)	1*	7.17 (d, *J* = 1.3 Hz)
2	7.92 (s)	2	7.80 (s)	2*	7.52 (d, *J* = 1.4 Hz)
3	7.73 (d, *J* = 8.3 Hz)	3	7.46 (s)	3*	7.46 (s)
4	7.83 (d, *J* = 8.4 Hz)	4	7.27 (s)	4*	7.28 (s)
5	8.23 (s)	5	8.03 (s)	5*	7.60 (d, *J* = 1.2 Hz)
6	7.53 (s)	6	7.22 (s)	6*	6.95 (d, *J* = 1.2 Hz)
7	7.49 (d, *J* = 8.1 Hz)	7	7.28 (d, *J* = 8.4 Hz)	7*	7.17 (d, *J* = 8.2 Hz)
8	6.25 (d, *J* = 8.0 Hz)	8	6.86 (d, *J* = 8.3 Hz)	8*	5.77 (d, *J* = 8.2 Hz)
9	7.71 (d, *J* = 8.3 Hz)	9	6.55 (d, *J* = 8.4 Hz)	9*	7.78 (d, *J* = 8.2 Hz)
10	6.90 (d, *J* = 8.3 Hz)	10	6.46 (d, *J* = 8.3 Hz)	10*	6.65 (d, *J* = 8.3 Hz)
11	6.92 (s)	11	7.12 (s)	11*	7.27 (s)
12	7.73 (s)	12	6.89 (d, *J* = 1.3 Hz)	12*	8.32 (s)
13	7.00 (d, *J* = 8.1 Hz)	13	6.55 (d, *J* = 8.4 Hz)	13*	8.06 (d, *J* = 8.1 Hz)
14	7.40 (d, *J* = 8.0 Hz)	14	6.78 (d, *J* = 8.3 Hz)	14*	7.97 (d, *J* = 8.2 Hz)
15	8.01 (s)	15	8.21 (d, *J* = 1.4 Hz)	15*	8.27 (d, *J* = 1.2 Hz)
16	7.26 (s)	16	7.42 (d, *J* = 1.3 Hz)	16*	7.89 (d, *J* = 1.3 Hz)
17	6.39 (d, *J* = 8.3 Hz)	17	6.89 (d, *J* = 8.3 Hz)	17*	7.27 (d, *J* = 8.1 Hz)
18	7.18 (d, *J* = 8.3 Hz)	18	7.81 (d, *J* = 8.3 Hz)	18*	7.33 (d, *J* = 8.3 Hz)
19	6.90 (d, *J* = 8.2 Hz)	19	7.92 (d, *J* = 8.3 Hz)	19*	7.58 (d, *J* = 8.4 Hz)
20	6.34 (d, *J* = 8.2 Hz)	20	7.44 (d, *J* = 8.1 Hz)	20*	7.45 (d, *J* = 8.1 Hz)
21	6.56 (s)	21	6.20 (s)		
22	6.92 (d, *J* = 8.0 Hz)	22	7.24 (d, *J* = 7.9 Hz)	22*	7.24 (d, *J* = 7.9 Hz)
23	7.40 (d, *J* = 8.1 Hz)	23	7.53 (d, *J* = 7.8 Hz)		
i	8.66 (s)	i	8.58 (s)	i*	7.00 (s)
t-Bu1	1.52 (s)	*t*-Bu1	1.41 (s)	*t*-Bu1*	1.44 (s)
t-Bu2	1.62 (s)	*t*-Bu2	1.65 (s)	*t*-Bu2*	1.14 (s)
t-Bu3	1.49 (s)	*t*-Bu3	0.86 (s)	*t*-Bu3*	1.74 (s)
t-Bu4	1.72 (s)	*t*-Bu4	1.73 (s)	*t*-Bu4*	1.64 (s)
		OHa	8.51 (d, *J* = 12.5 Hz)	Ha	6.69 (dd, *J* = 12.5, 4.8 Hz)
		OHb	9.65 (d, *J* = 9.2 Hz)	Hb	6.41
		OHc	7.13 (s)	Hc	5.59
		OHe	7.78 (s)	Hd	5.21 (dd, *J* = 8.8, 7.0 Hz)
		OHf	7.06 (t, *J* = 4.1 Hz)	He	5.51
		Hf	5.43	Hf	5.55

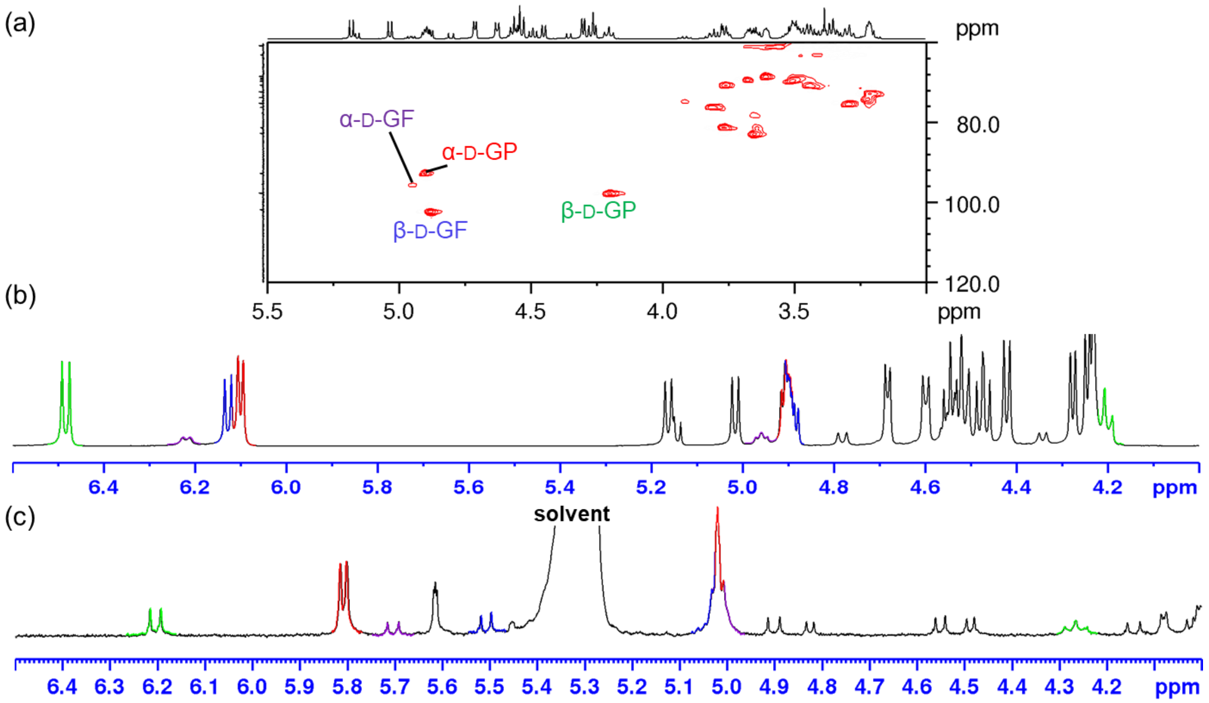

Supplementary Figure 7. (a,b) ^{1}H-^{13}C HSQC and partial ^{1}H NMR (400 MHz, 25 °C) spectra of D-galactose (10 mM) in DMSO-d_6. (c) Partial ^{1}H NMR spectrum (400 MHz, 25 °C) of D-galactose (1.2 mM) after 24 h standing at room temperature in 10% (v/v) DMSO-d_6/CD_2Cl_2. α-D-GP, β-D-GP, α-D-GF, and β-D-GF are highlighted with red, green, purple, and blue, respectively.

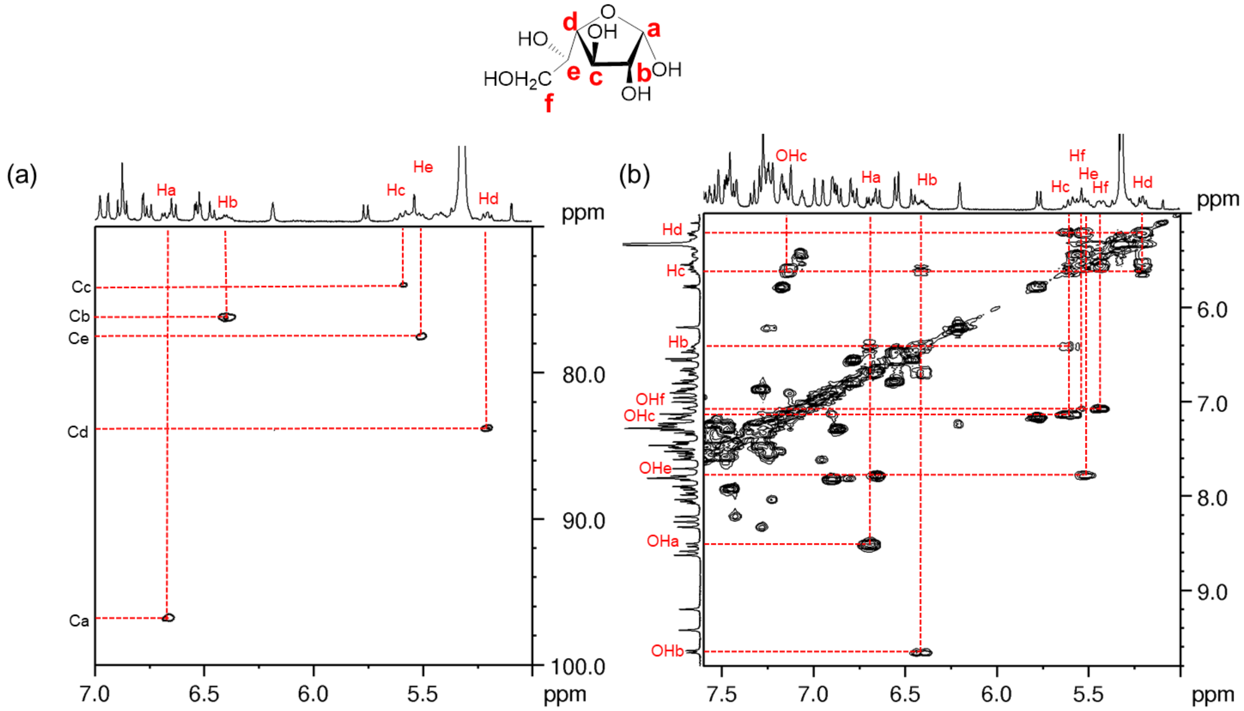

Supplementary Figure 8. (a) ^{1}H-^{13}C HSQC and (b) COSY spectra (400 MHz, 25 °C) of **4**⊃α-D-GF·H_2O (4 mM) in CD_2Cl_2.

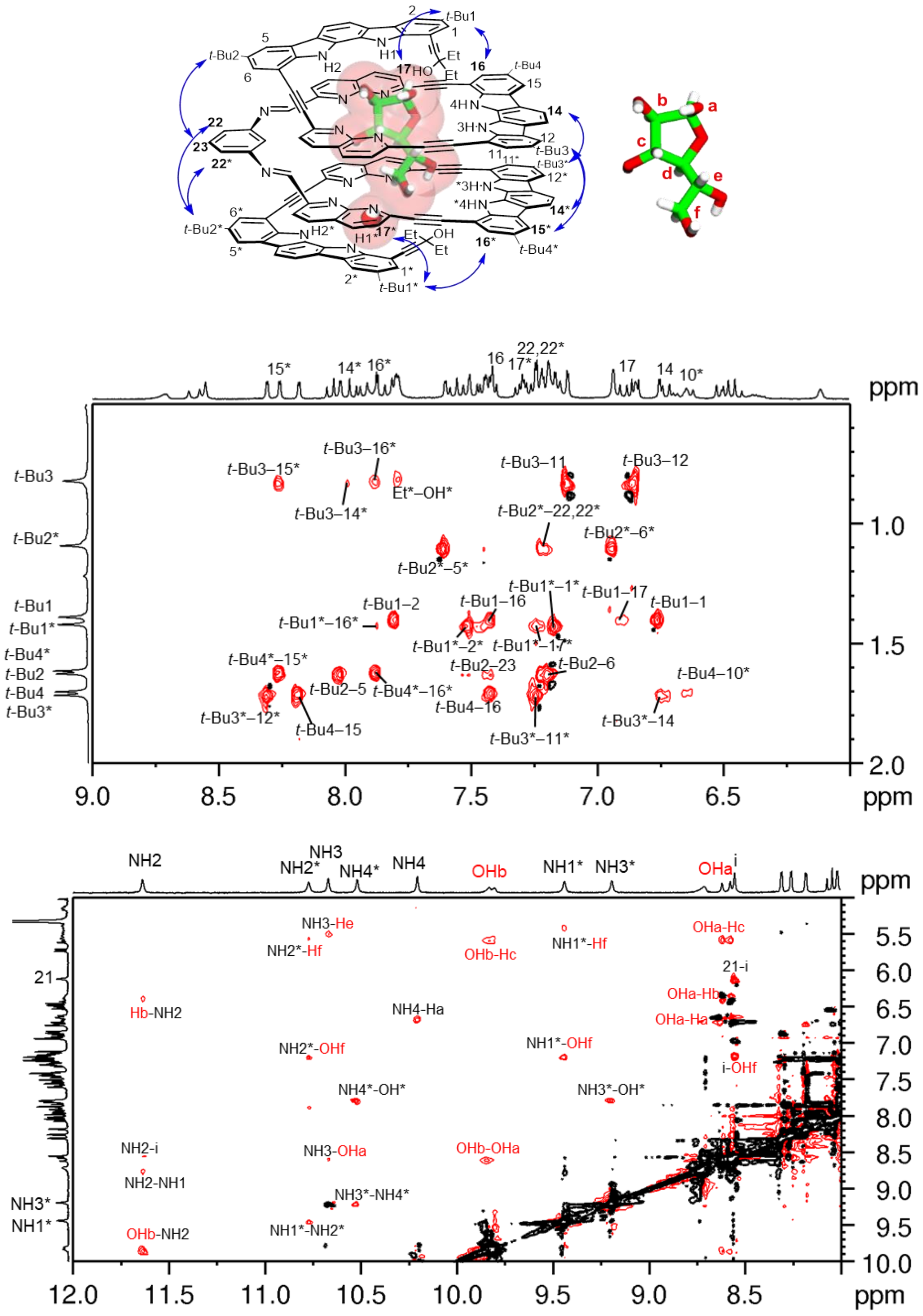

Supplementary Figure 9. Partial ^{1}H-^{1}H ROESY spectrum (300 MHz, 0 °C, mixing time: 100 ms) of **4**⊃α-D-GF·H_2O (5.0 mM) in CD_2Cl_2. ^{1}H NMR signals for α-D-GF are depicted in red.

3.2 ^{1}H NMR spectrum of **6** with the peak assignment

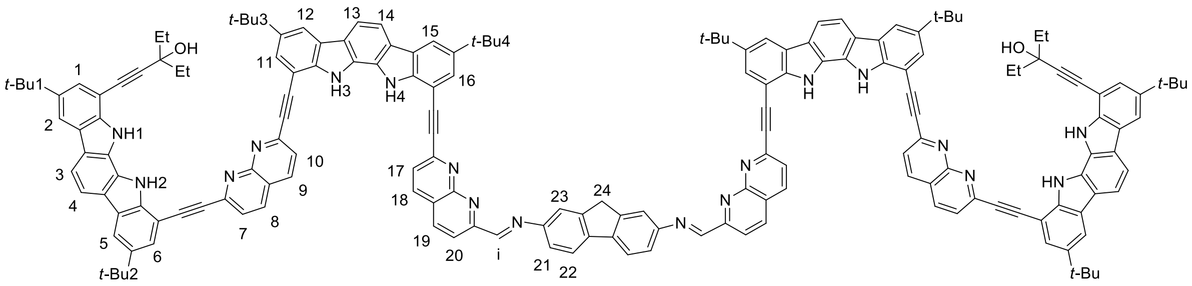

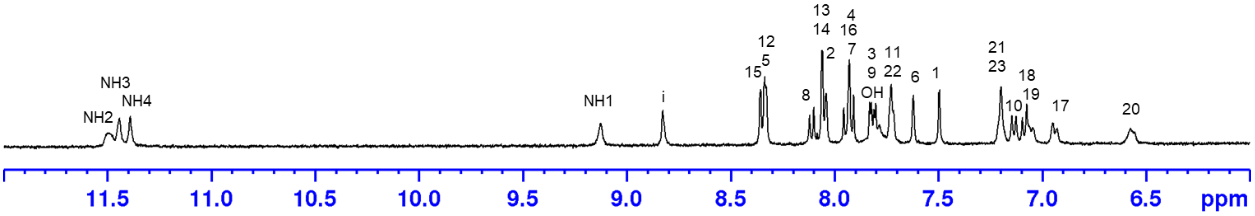

Supplementary Figure 10. Partial ^{1}H NMR spectrum (400 MHz, 25 °C) of **6** in 1% (v/v) MeOH-d_3/CD_2Cl_2.

Supplementary Table 3. ^{1}H NMR chemical shifts (400 MHz, 25 °C) of **6** in 1% (v/v) MeOH-d_3/CD_2Cl_2.

Atom	^{1}H (ppm)	Atom	^{1}H (ppm)
NH1	9.12 (s)	14	8.06 (s)
NH2	11.50 (s)	15	8.36 (s)
NH3	11.44 (s)	16	7.93 (s)
NH4	11.39 (s)	17	6.93 (d, J = 8.4 Hz)
1	7.50 (s)	18	7.05 (d, J = 8.2 Hz)
2	8.04 (s)	19	7.03 (d, J = 8.2 Hz)
3	7.82 (d, J = 8.2 Hz)	20	6.54 (d, J = 8.1 Hz)
4	7.92 (d, J = 8.1 Hz)	21	7.19 (d, J = 7.9 Hz)
5	8.35 (s)	22	7.71 (d, J = 7.9 Hz)
6	7.61 (s)	23	7.20 (s)
7	7.92 (d, J = 8.1 Hz)	24	3.33 (s)
8	8.11 (s)	i	8.83 (s)
9	7.78 (d, J = 8.3 Hz)	*t*-Bu1	1.60 (s)
10	7.12 (d, J = 8.2 Hz)	*t*-Bu2	1.65 (s)
11	7.73 (s)	*t*-Bu3	1.56 (s)
12	8.33 (s)	*t*-Bu4	1.53 (s)
13	8.06 (s)		

3.3 ^{1}H NMR and ^{1}H-^{1}H ROESY spectra of (**6**-*MP*)$_2$⊃(me-β-D-glc)$_2$

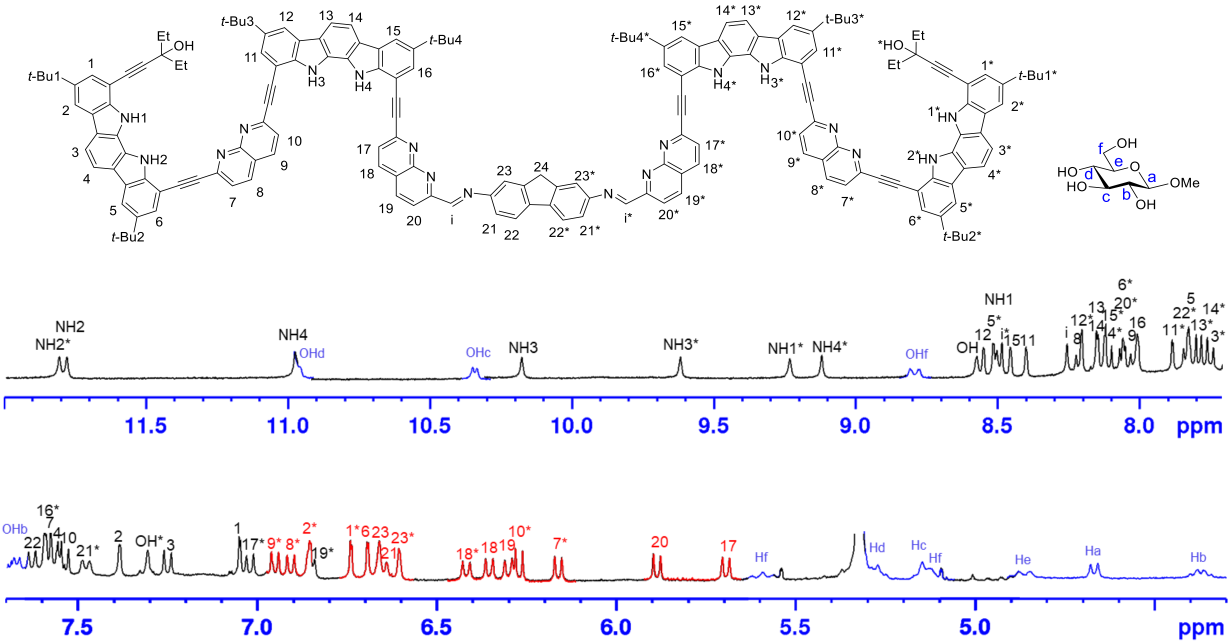

Supplementary Figure 11. Partial ^{1}H NMR spectrum (400 MHz, 25 °C) of (**6**-*MP*)$_2$⊃(me-β-D-glc)$_2$ in 2% (v/v) DMSO-d_6/CD_2Cl_2. The CH signals of stacked aryl planes are depicted in red, and me-β-D-glc signals are in blue.

Supplementary Table 4. ^{1}H NMR chemical shifts (400 MHz, 25 °C) of (**6**-*MP*)$_2$⊃(me-β-D-glc)$_2$ in 2% (v/v) DMSO-d_6/CD_2Cl_2.

Atom	^{1}H (ppm)	Atom	^{1}H (ppm)
NH1	8.50 (s)	NH1*	9.24 (s)
NH2	11.78 (s)	NH2*	11.81 (s)
NH3	10.18 (s)	NH3*	9.62 (s)
NH4	10.98 (s)	NH4*	9.12 (s)
1	7.05 (d, *J* = 1.5 Hz)	1*	6.74 (d, *J* = 1.5 Hz)
2	7.38 (d, *J* = 1.5 Hz)	2*	6.85 (d, *J* = 1.4 Hz)
3	7.25 (d, *J* = 8.1 Hz)	3*	7.75 (d, *J* = 8.2 Hz)
4	7.55 (d, *J* = 8.1 Hz)	4*	8.11 (d, *J* = 8.2 Hz)
5	7.83 (s)	5*	8.52 (d, *J* = 1.3 Hz)
6	6.69 (d, *J* = 1.5 Hz)	6*	8.06 (d, *J* = 1.4 Hz)
7	7.58 (d, *J* = 8.0 Hz)	7*	6.16 (d, *J* = 7.9 Hz)
8	8.22 (d, *J* = 8.0 Hz)	8*	6.91 (d, *J* = 8.1 Hz)
9	8.02 (d, *J* = 8.1 Hz)	9*	6.95 (d, *J* = 8.1 Hz)
10	7.54 (d, *J* = 8.0 Hz)	10*	6.27 (d, *J* = 8.1 Hz)
11	8.40 (d, *J* = 1.3 Hz)	11*	7.88 (d, *J* = 1.5 Hz)
12	8.55 (d, *J* = 1.4 Hz)	12*	8.20 (s)
13	8.16 (d, *J* = 8.1 Hz)	13*	7.79 (d, *J* = 8.0 Hz)

14	8.14 (d, *J* = 8.1 Hz)	14*	7.77 (d, *J* = 8.2 Hz)
15	8.46 (s)	15*	8.12 (s)
16	8.01 (s)	16*	7.59 (s)
17	5.70 (d, *J* = 8.0 Hz)	17*	7.02 (d, *J* = 7.9 Hz)
18	6.36 (d, *J* = 8.0 Hz)	18*	6.42 (d, *J* = 8.2 Hz)
19	6.30 (d, *J* = 8.0 Hz)	19*	6.85 (d, *J* = 8.0 Hz)
20	5.89 (d, *J* = 8.0 Hz)	20*	8.06 (d, *J* = 8.1 Hz)
21	6.65 (d, *J* = 7.8 Hz)	21*	7.48 (d, *J* = 7.9 Hz)
22	7.63 (d, *J* = 7.9 Hz)	22*	7.84 (d, *J* = 8.0 Hz)
23	6.66 (s)	23*	6.61 (s)
24	3.43 (s)	24*	3.38 (s)
i	8.26 (s)	i*	8.49 (s)
t-Bu1	1.33 (s)	*t*-Bu1*	0.83 (s)
t-Bu2	1.04 (s)	*t*-Bu2*	1.92 (s)
t-Bu3	1.90 (s)	*t*-Bu3*	1.76 (s)
t-Bu4	1.82 (s)	*t*-Bu4*	1.66 (s)
OMe	1.42 (s)	Ha	4.67 (d, *J* = 8.1 Hz)
OHb	7.67 (d, *J* = 6.2 Hz)	Hb	4.37
OHc	10.34 (d, *J* = 6.0 Hz)	Hc	5.15
OHd	10.97 (d, *J* = 8.1 Hz)	Hd	5.27
OHf	8.80 (d, *J* = 10.6 Hz)	He	4.86
Hf	5.59	Hf	5.12

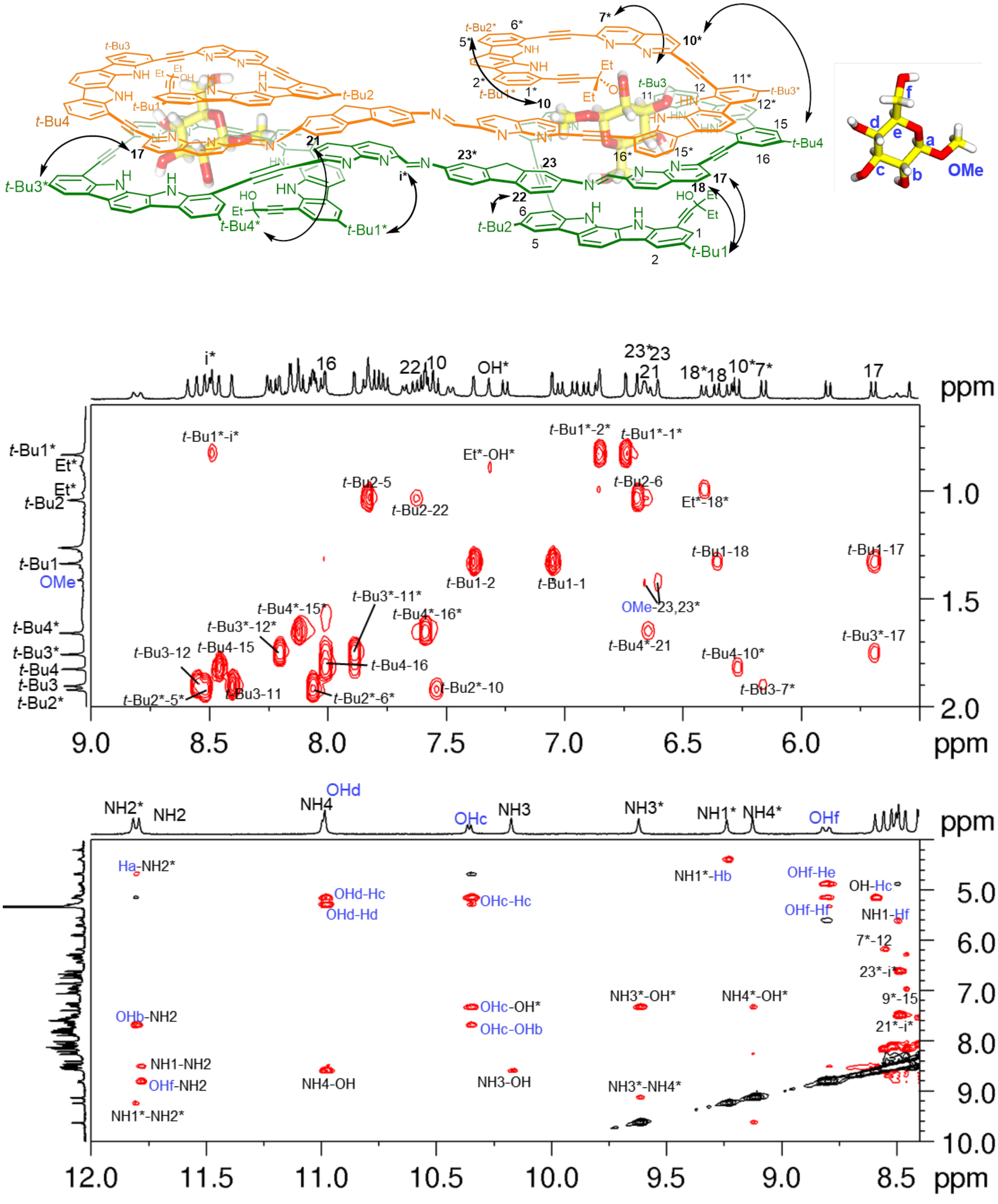

Supplementary Figure 12. Partial ^{1}H-^{1}H ROESY spectrum (400 MHz, 25 °C, mixing time: 400 ms) of (**6**-*MP*)$_2$⊃(me-β-D-glc)$_2$ (4.0 mM) in 2% (v/v) DMSO-d_6/CD_2Cl_2. ^{1}H NMR signals for me-β-D-glc are depicted in blue.

3.4 ^{1}H NMR and ^{1}H-^{1}H ROESY spectra of (**6**-*MM*)$_2$⊃(me-β-D-gal·2H_2O)$_2$

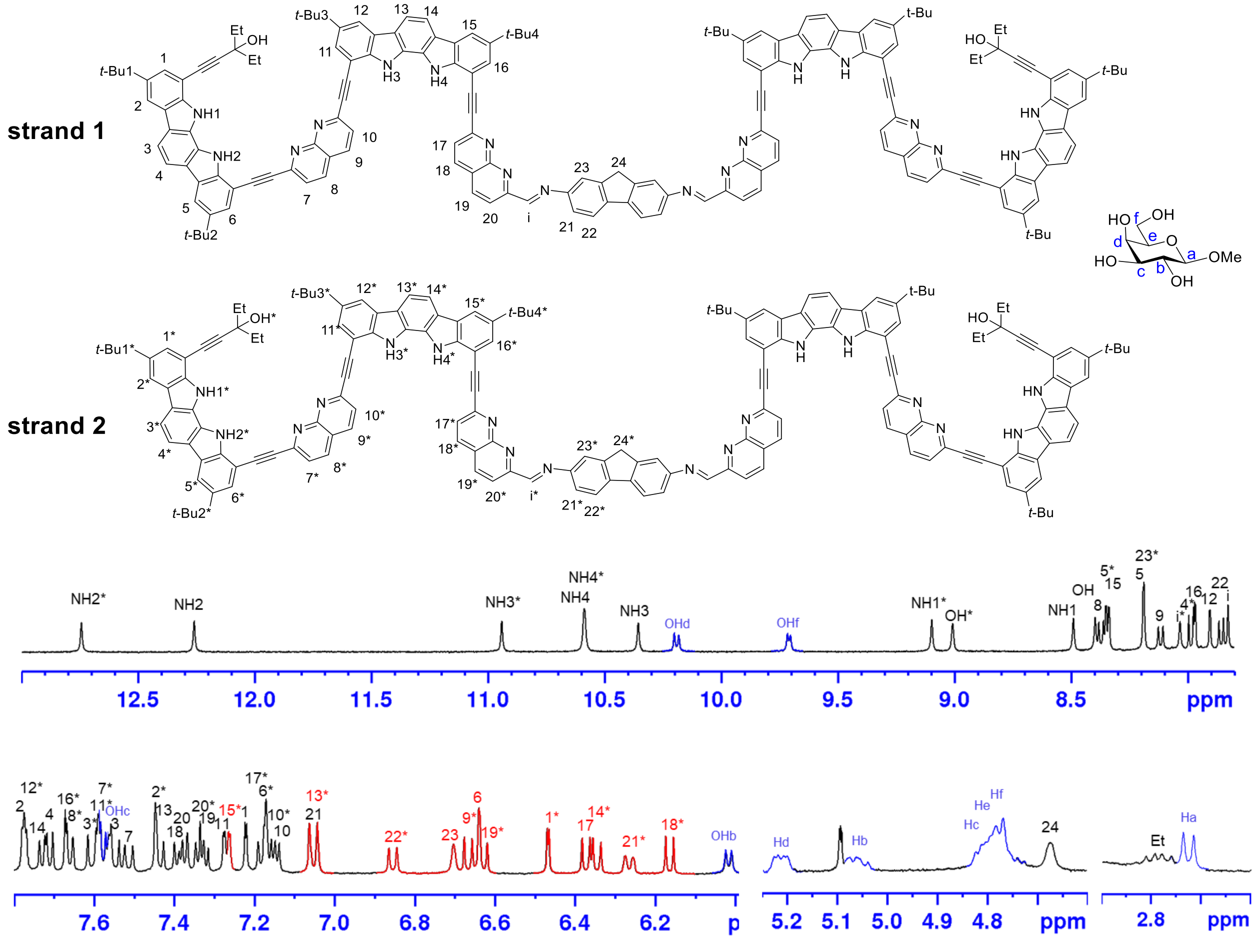

Supplementary Figure 13. Partial ^{1}H NMR spectrum (400 MHz, 25 °C) of (**6**-*MM*)$_2$⊃(me-β-D-gal·2H_2O)$_2$ in 2% (v/v) DMSO-d_6/CD_2Cl_2. The CH signals of stacked aryl planes are depicted in red, and me-β-D-gal signals are in blue.

Supplementary Table 5. ^{1}H NMR chemical shifts (400 MHz, 25 °C) of (**6**-*MM*)$_2$⊃(me-β-D-gal·2H_2O)$_2$ in 2% (v/v) DMSO-d_6/CD_2Cl_2.

Atom	^{1}H (ppm)	Atom	^{1}H (ppm)
NH1	8.49 (s)	NH1*	9.10 (s)
NH2	12.26 (s)	NH2*	12.74 (s)
NH3	10.36 (s)	NH3*	10.94 (s)
NH4	10.59 (s)	NH4*	10.59 (s)
1	7.22 (d, *J* = 1.5 Hz)	1*	6.47 (d, *J* = 1.6 Hz)
2	7.78 (d, *J* = 1.4 Hz)	2*	7.45 (d, *J* = 1.5 Hz)
3	7.55 (d, *J* = 8.1 Hz)	3*	7.60 (d, *J* = 8.2 Hz)
4	7.71 (d, *J* = 8.1 Hz)	4*	7.98 (d, *J* = 8.2 Hz)
5	8.19 (d, *J* = 1.4 Hz)	5*	8.35 (d, *J* = 1.5 Hz)
6	6.64 (s)	6*	7.17 (s)

7	7.51 (d, *J* = 8.1 Hz)	7*	7.58 (d, *J* = 8.3 Hz)
8	8.37 (d, *J* = 8.1 Hz)	8*	7.66 (d, *J* = 8.1 Hz)
9	8.11 (d, *J* = 8.3 Hz)	9*	6.67 (d, *J* = 8.1 Hz)
10	7.15 (d, *J* = 7.9 Hz)	10*	7.16 (d, *J* = 8.2 Hz)
11	7.27 (d, *J* = 1.5 Hz)	11*	7.59 (d, *J* = 1.2 Hz)
12	7.90 (d, *J* = 1.4 Hz)	12*	7.77 (d, *J* = 1.5 Hz)
13	7.44 (d, *J* = 8.2 Hz)	13*	7.05 (d, *J* = 8.1 Hz)
14	7.73 (d, *J* = 8.2 Hz)	14*	6.35 (d, *J* = 8.1 Hz)
15	8.34 (d, *J* = 1.3 Hz)	15*	7.26 (d, *J* = 1.5 Hz)
16	7.97 (d, *J* = 1.3 Hz)	16*	7.67 (d, *J* = 1.5 Hz)
17	6.37 (d, *J* = 8.1 Hz)	17*	7.18 (d, *J* = 8.1 Hz)
18	7.39 (d, *J* = 8.2 Hz)	18*	6.16 (d, *J* = 8.0 Hz)
19	7.32 (d, *J* = 8.2 Hz)	19*	6.63 (d, *J* = 8.1 Hz)
20	7.38 (d, *J* = 8.2 Hz)	20*	7.34 (d, *J* = 8.1 Hz)
21	7.05 (d, *J* = 8.1 Hz)	21*	6.26 (d, *J* = 8.0 Hz)
22	7.86 (d, *J* = 7.9 Hz)	22*	6.85 (d, *J* = 8.0 Hz)
23	6.70 (s)	23*	8.19 (s)
24	3.85 (s)	24*	4.67 (s)
i	7.83 (s)	i*	8.03 (s)
t-Bu1	1.56 (s)	*t*-Bu1*	1.20 (s)
t-Bu2	1.56 (s)	*t*-Bu2*	1.74 (s)
t-Bu3	1.49 (s)	*t*-Bu3*	1.11 (s)
t-Bu4	1.80 (s)	*t*-Bu4*	1.66 (s)
OMe	2.38 (s)	Ha	2.73 (d, *J* = 8.1 Hz)
OHb	6.02 (d, *J* = 5.9 Hz)	Hb	5.06
OHc	7.56	Hc	4.81
OHd	10.19 (d, *J* = 7.8 Hz)	Hd	5.21
OHf	9.71 (d, *J* = 5.8 Hz)	He	4.78
Hf	4.74	Hf	4.74

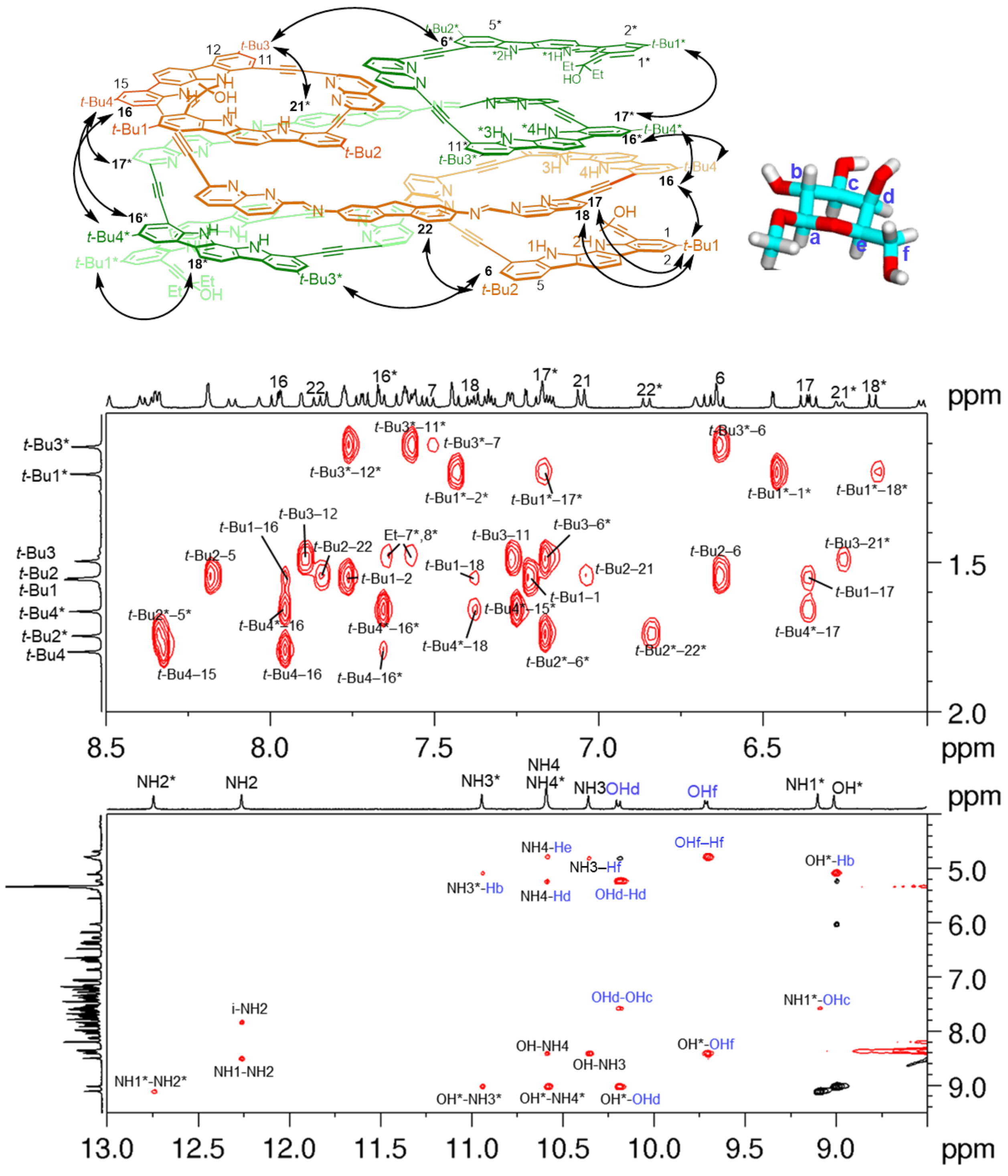

Supplementary Figure 14. Partial ^{1}H-^{1}H ROESY spectrum (400 MHz, 25 °C, mixing time: 400 ms) of (**6**-*MM*)$_2$⊃(me-β-D-gal·2H_2O)$_2$ (5.0 mM) in 2% (v/v) DMSO-d_6/CD_2Cl_2. ^{1}H NMR signals for me-β-D-gal are depicted in blue.

3.5 Time-dependent ^{1}H NMR spectra for a 1:1 mixture of **4** and D-galactose

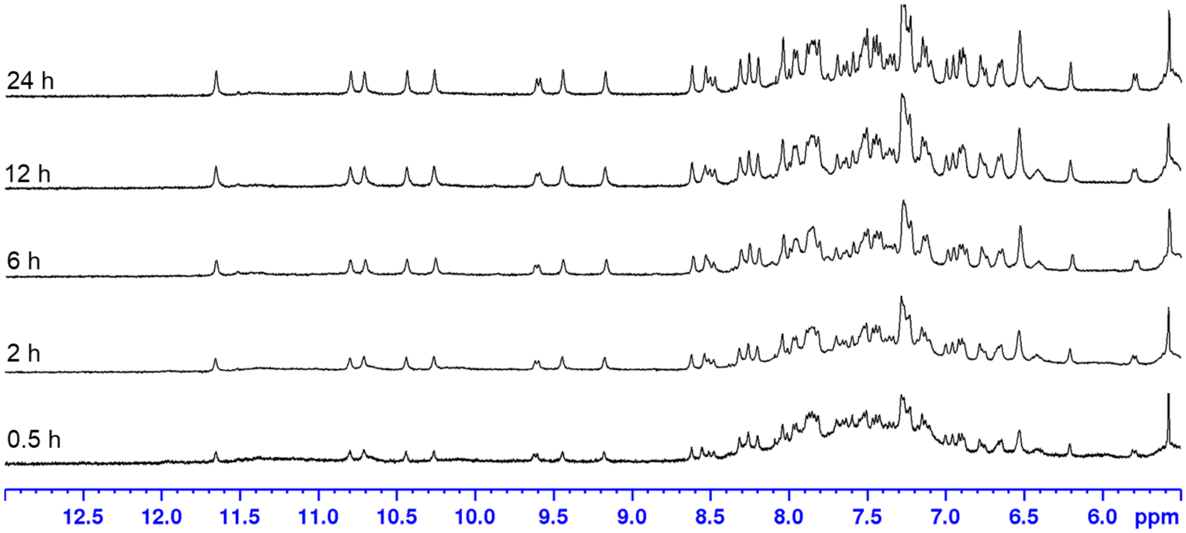

Supplementary Figure 15. Time-dependent ^{1}H NMR spectra (400 MHz, 25 °C) of a mixture of **4** (1.0 mM) and D-galactose (1.1 equiv.) in 10% (v/v) DMSO-d_6/CD_2Cl_2. Here, D-galactose was used after 24 h standing in 10% (v/v) DMSO-d_6/CD_2Cl_2 at room temperature. 1,2-Dichloroethane and dimethyl sulfoxide were used as internal standards.

3.6 ^{1}H NMR spectra of **4** and **6** in the presence of mixed guests

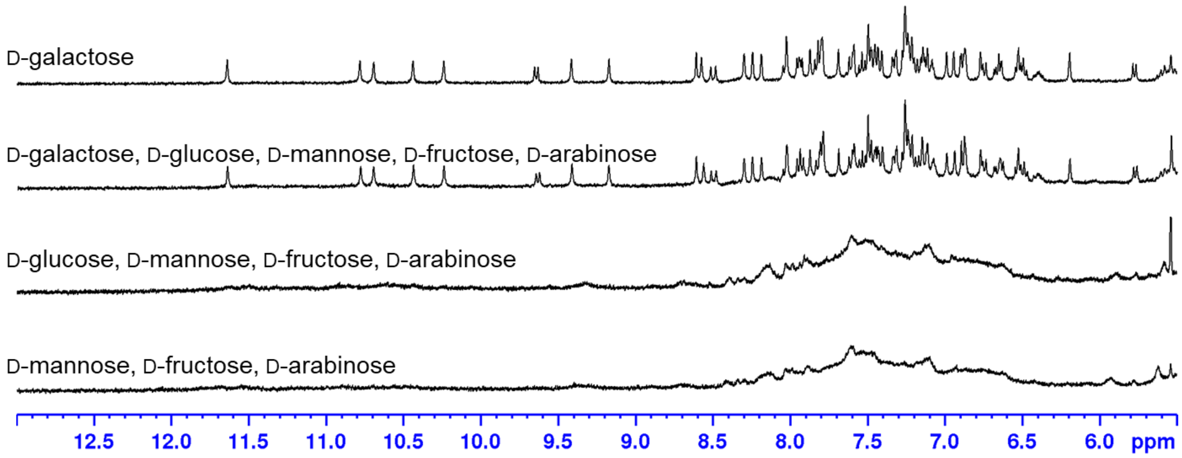

Supplementary Figure 16. ^{1}H NMR spectra (400 MHz, 25 °C) of a **4** (1.0 mM) in the presence of mixed guests (3 equiv. of each guest) in 5% (v/v) DMSO-d_6/CD_2Cl_2.

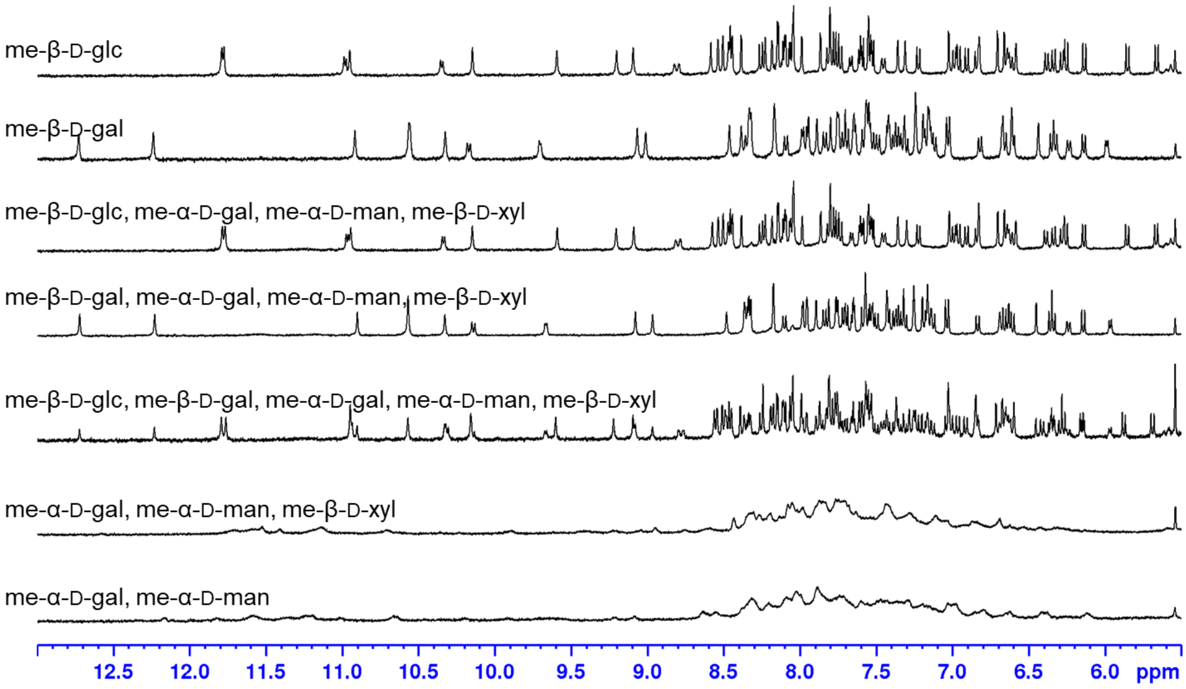

Supplementary Figure 17. ^{1}H NMR spectra (400 MHz, 25 °C) of a **6** (1.0 mM) in the presence of mixed guests (2.5 equiv. of each guest) in 5% (v/v) DMSO-d_6/CD_2Cl_2.

3.7 Temperature-dependent ^{1}H NMR spectra for a mixture of **6**, me-β-D-gal, and me-β-D-glc

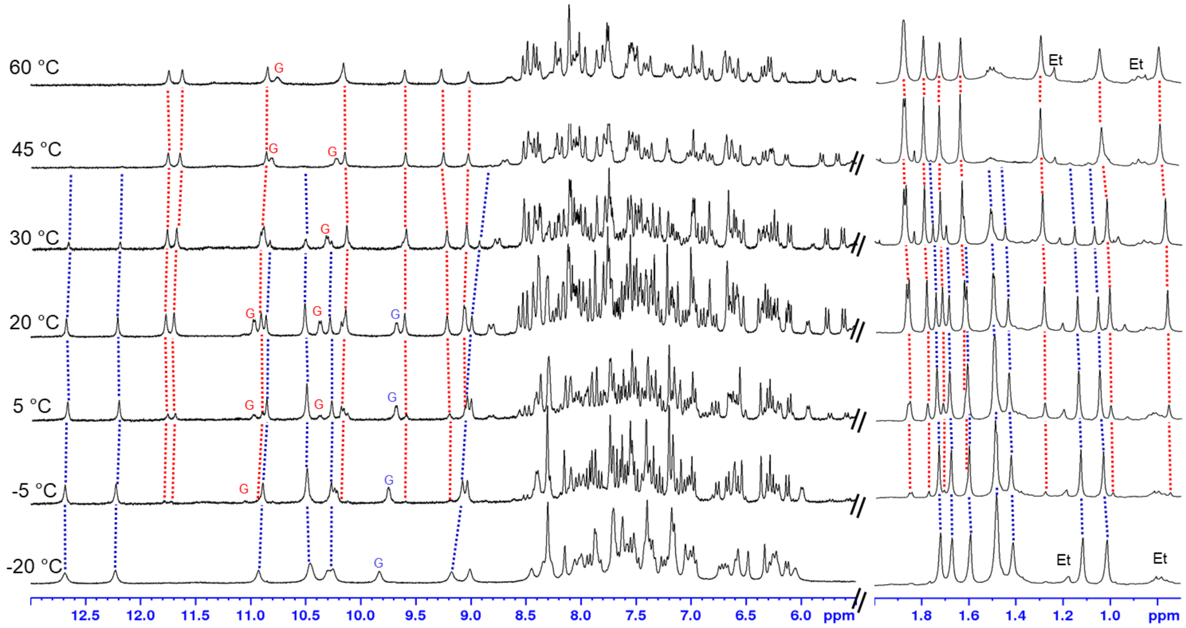

Supplementary Figure 18. Partial ^{1}H NMR spectra (300 MHz) of **6** (1.00 mM), me-β-D-gal (3 equiv.), and me-β-D-glc (3 equiv.) in 5% (v/v) DMSO-d_6/$(CD_2Cl)_2$ (containing 0.04–0.06% water) at various temperatures. The NH and *t*-Bu signals of (**6**-*MM*)$_2$⊃(me-β-D-gal·2H_2O)$_2$ and (**6**-*MP*)$_2$⊃(me-β-D-glc)$_2$ are marked by dashed blue and red lines, respectively. The signals for ethyl groups at the end of diimine **6** are marked as Et's. The signals of bound me-β-D-gal and me-β-D-glc are marked as blue and red G, respectively.

4. CD spectra of 4

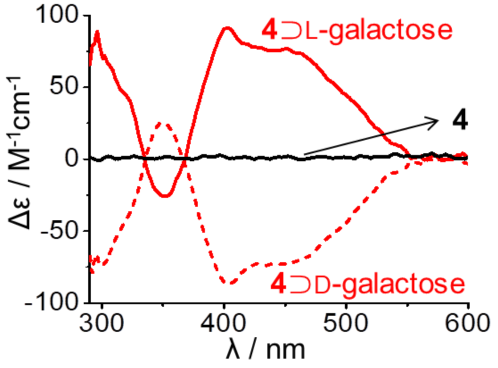

Supplementary Figure 19. CD spectra of **4** (2.00×10^{-5} M, 25 ± 1 °C) in the absence and presence of excess D- and L-galactose (~50 equiv.) in 10% (v/v) DMSO/CH_2Cl_2.

5. Binding studies

5.1 CD titrations of **4** with various guests

Diimine **4** = H, guest = G

$$H + G \rightleftarrows HG \qquad K_{obs} = \frac{[H \cdot G]}{[H][G]}$$

Supplementary Table 6. Observed association constants (K_{obs}, M^{-1})[a] between **4** and guests in 10% DMSO/CH_2Cl_2 (containing 0.04–0.06% water)

Entry	Guest	K_{obs} (M^{-1})
1	D-galactose	54000 (±1700)
2	D-glucose[b]	~4000
3	D-mannose	4580 (±220)
4	D-fructose	3020 (±240)
5	D-arabinose	2490 (±50)

[a] The observed association constants were estimated by nonlinear squares fitting, assuming 1:1 binding modes. [b] Induced CD signal changes upon binding were too small to accurately calculate the association constant. All titrations were duplicated at 25 ± 1 °C.

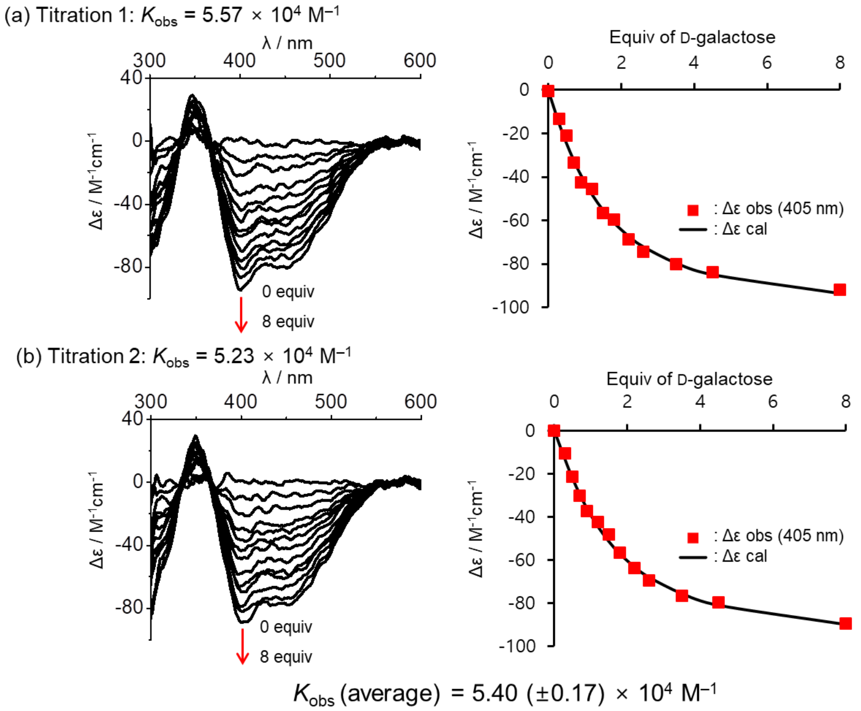

Supplementary Figure 20. (a,b) CD spectral changes of **4** (2.00 × 10^{-5} M) with increasing the amount of D-galactose in 10% (v/v) DMSO/CH_2Cl_2 (containing 0.04–0.06% water) at 25 ± 1 °C, and experimental data (dots) and theoretical curves (lines) are shown right.

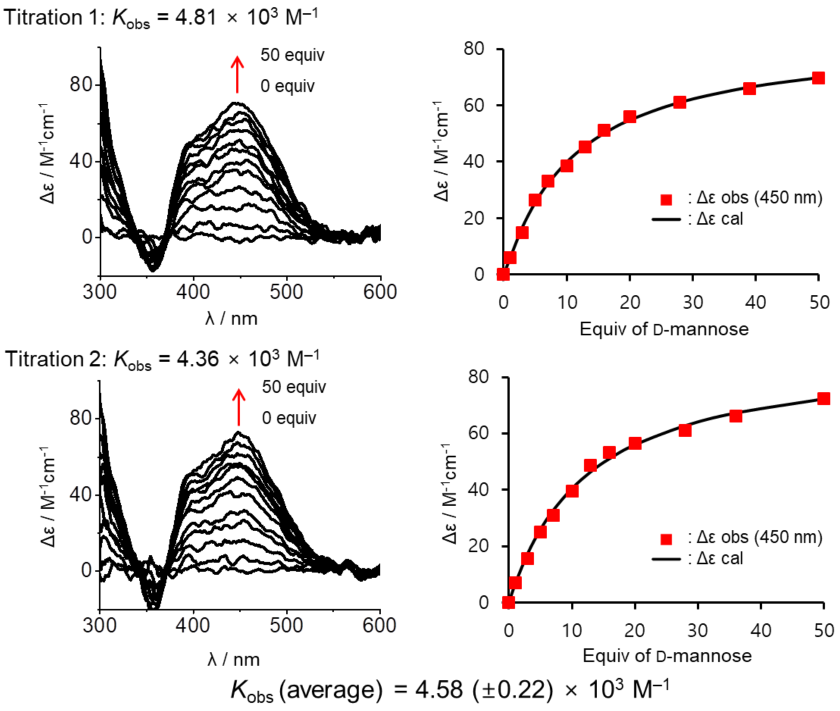

Supplementary Figure 21. (a,b) CD spectral changes of **4** (2.00 × 10^{-5} M) with increasing the amount of D-mannose in 10% (v/v) DMSO/CH_2Cl_2 (containing 0.04–0.06% water) at 25 ± 1 °C, and experimental data (dots) and theoretical curves (lines) are shown right.

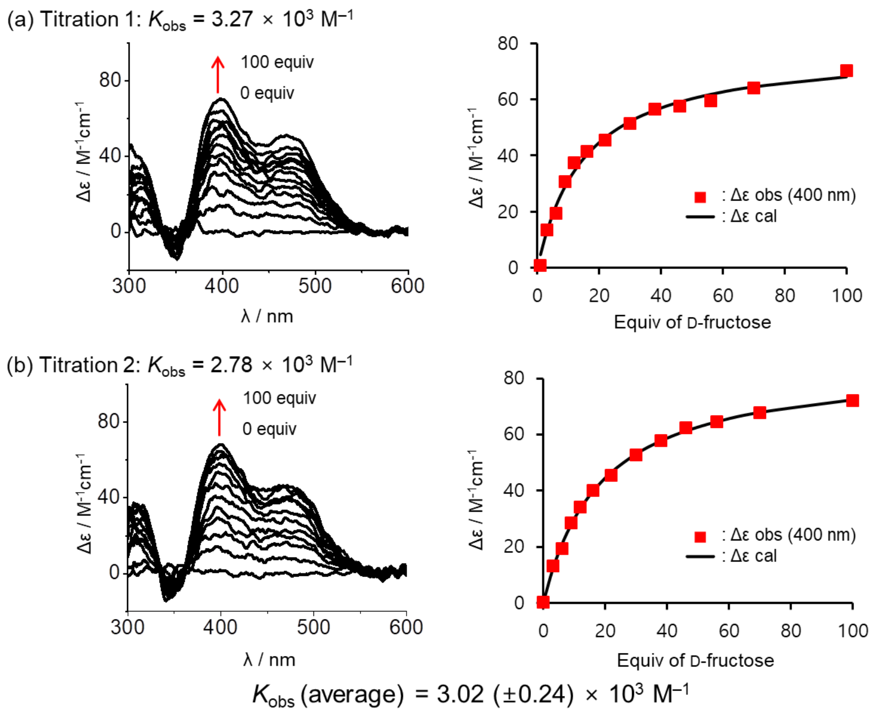

Supplementary Figure 22. (a,b) CD spectral changes of **4** (2.00×10^{-5} M) with increasing the amount of D-fructose in 10% (v/v) DMSO/CH_2Cl_2 (containing 0.04–0.06% water) at 25 ± 1 °C, and experimental data (dots) and theoretical curves (lines) are shown right.

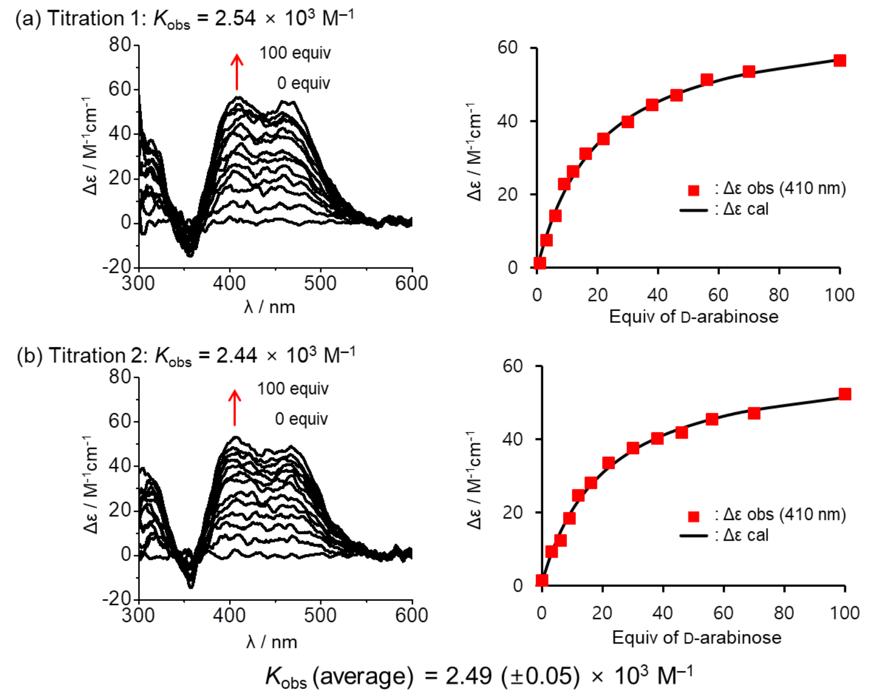

Supplementary Figure 23. (a,b) CD spectral changes of **4** (2.00×10^{-5} M) with increasing the amount of D-arabinose in 10% (v/v) DMSO/CH_2Cl_2 (containing 0.04–0.06% water) at 25 ± 1 °C, and experimental data (dots) and theoretical curves (lines) are shown right.

5.2 ^{1}H NMR titrations of **6** with me-β-D-glc and me-β-D-gal

Stock solutions of **6** (1.00 mM or 1.50 mM) and each guest (5.00 mM) were prepared separately in 5% (v/v) DMSO-d_6/CD_2Cl_2 (containing 0.04–0.06% water). A 400 μL of the **6** solution was taken in a NMR tube and an initial spectrum was taken to determine the chemical shifts of unbounded diimine **6**. Aliquots of the guest solution were added to the NMR tube and the spectrum was recorded after each addition.

Diimine **6** = H, guest = G

$$2\ \mathrm{H} + 2\ \mathrm{G} \rightleftarrows (\mathrm{H})_2 \cdot (\mathrm{G})_2 \qquad K = \frac{[\mathrm{H}_2 \cdot \mathrm{G}_2]}{[\mathrm{H}]^2[\mathrm{G}]^2}$$

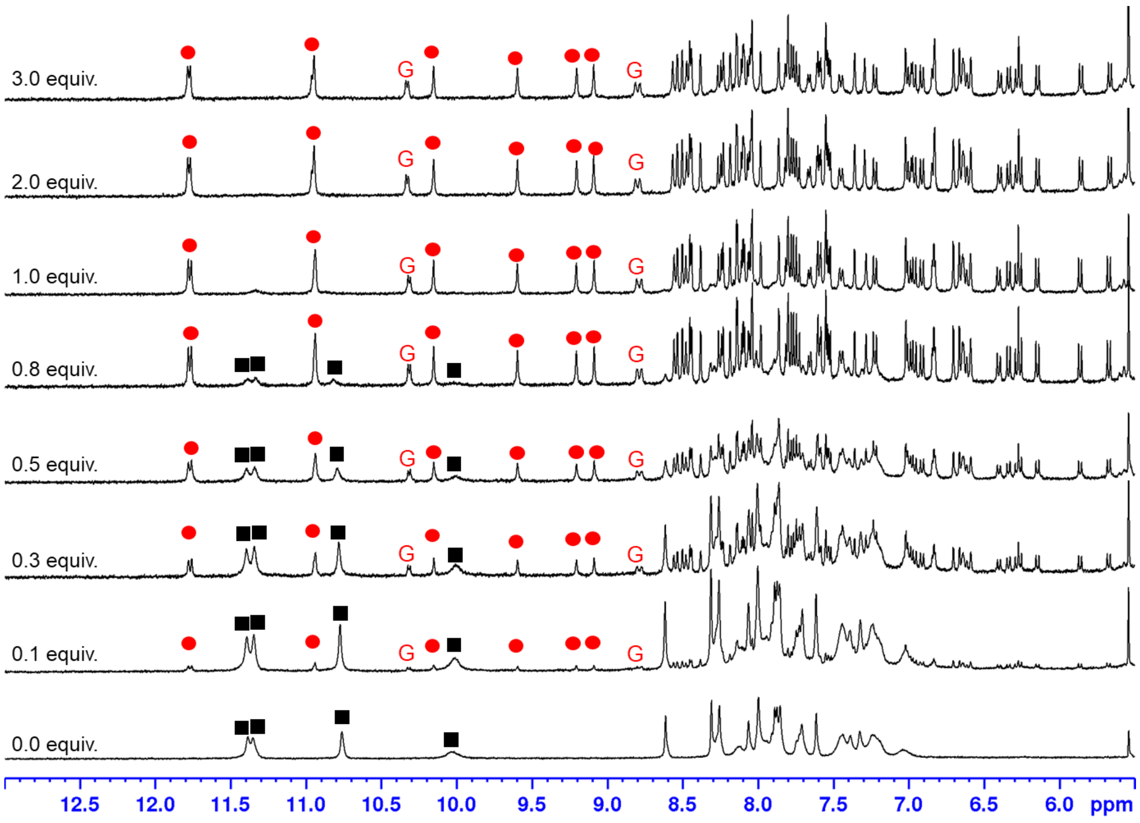

Supplementary Figure 24. Partial ^{1}H NMR spectral changes of **6** (1.00 mM, 25 ± 1 °C) with increasing the amount of me-β-D-glc in 5% (v/v) DMSO-d_6/CD_2Cl_2 (containing 0.04–0.06% water). The NH signals of unbound **6** and its 2:2 complex are marked with black squares and red circles, respectively. The signals of bound me-β-D-glc are marked as G.

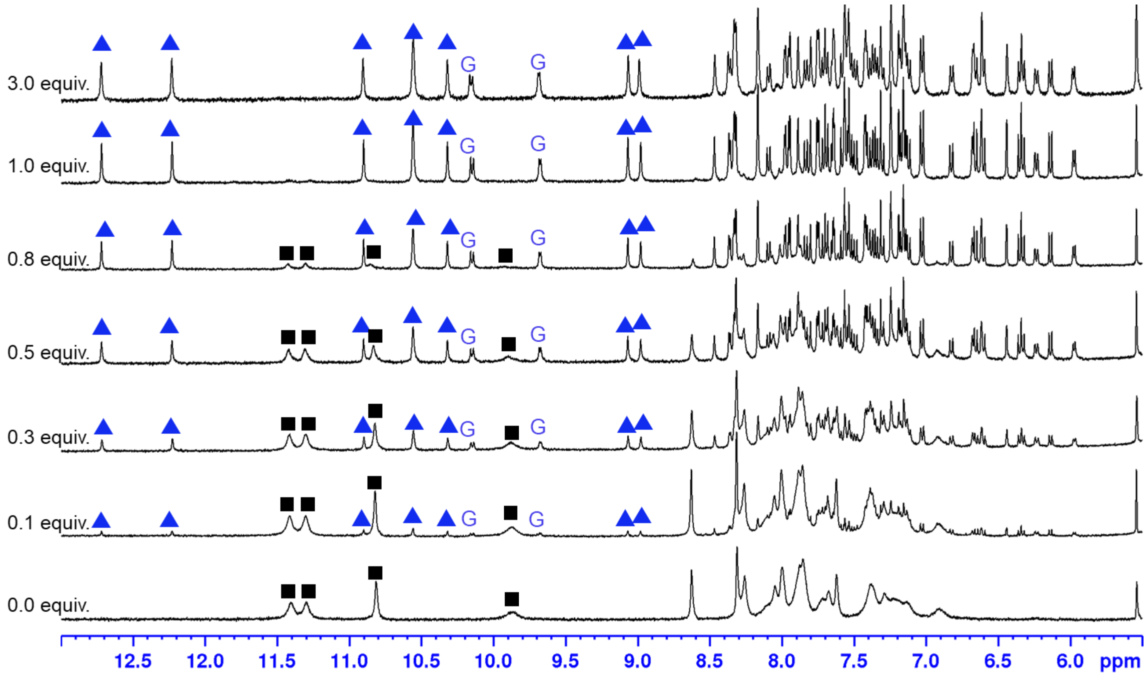

Supplementary Figure 25. Partial ^{1}H NMR spectral changes of **6** (1.50 mM, 25 ± 1 °C) with increasing the amount of me-β-D-gal in 5% (v/v) DMSO-d_6/CD_2Cl_2 (containing 0.04–0.06% water). The NH signals of unbound **6** and its 2:2 complex are marked with black squares and blue triangles, respectively. The signals of bound me-β-D-gal are marked as G.

5.3 CD titrations of 6 with me-β-D-gal

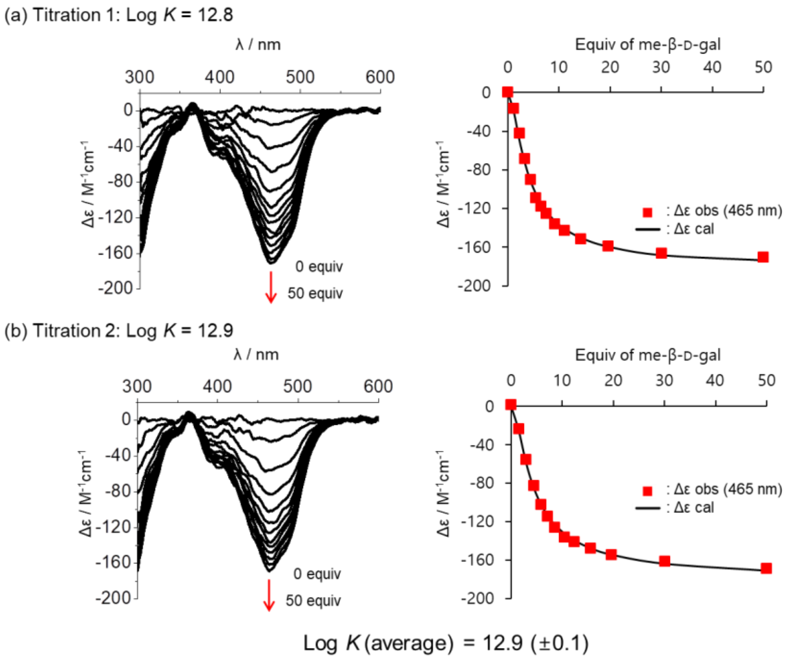

Supplementary Figure 26. (a,b) CD spectral changes of **6** (2.00×10^{-5} M) with increasing the amount of me-β-D-gal in 5% (v/v) DMSO/CH_2Cl_2 (containing 0.04–0.06% water) at 25 ± 1 °C, and experimental data (dots) and theoretical curves (lines) are shown right.

5.4 UV-visible titrations of **6** with me-β-D-glc and me-β-D-gal

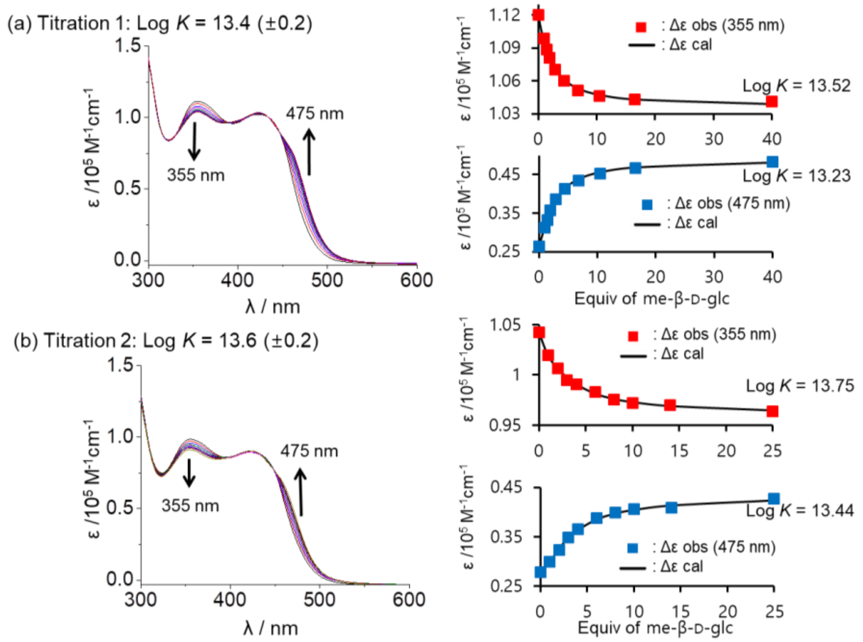

Log *K* (average) = 13.5 (±0.1)

Supplementary Figure 27. (a,b) UV-visible absorption spectral changes of **6** (2.00×10^{-5} M) with increasing the amount of me-β-D-glc in 5% (v/v) DMSO/CH_2Cl_2 (containing 0.04–0.06% water) at 25 ± 1 °C, and experimental data (dots) and theoretical curves (lines) are shown right. In (a,b), log *K's* are reported as mean values (± standard deviation).

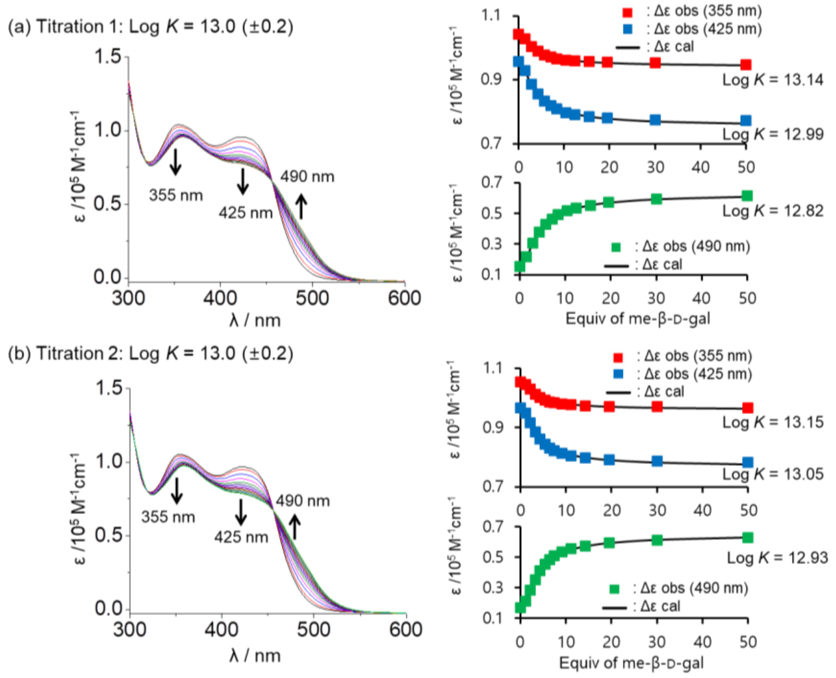

Log *K* (average) = 13.0 (±0.1)

Supplementary Figure 28. (a,b) UV-visible absorption spectral changes of **6** (2.00×10^{-5} M) with increasing the amount of me-β-D-gal in 5% (v/v) DMSO/CH_2Cl_2 (containing 0.04–0.06% water) at 25 ± 1 °C, and experimental data (dots) and theoretical curves (lines) are shown right. In (a,b), log *K's* are reported as mean values (± standard deviation).

5.5 Isothermal titration calorimetry (ITC) experiments for binding **6** with various guests

Supplementary Table 7. Titration results between **6** and methyl glycoside guests in 5% DMSO/CH_2Cl_2 (containing 0.04–0.06% water)

Entry	Guest	Log *K* (2:2 complex)	Δ*G*° (kJ mol^{-1})	Δ*H*° (kJ mol^{-1})	TΔ*S*° (kJ mol^{-1})
1	me-β-D-glc	13.5 (±0.1)	−76.4	−56.0	+20.4
2	me-β-D-gal	13.2 (±0.1)	−74.4	−130.2	−55.8
3	me-β-D-xyl	10.1 (±0.2)	−57.2	−38.9	+18.3
4	me-α-D-gal	10.1 (±0.1)	−57.2	−42.2	+15.0
5	me-α-D-man	9.9 (±0.2)	−56.0	−24.7	+31.3

K = [diimine$_2$·guest$_2$]/[diimine]^2[guest]^2. All titrations were duplicated at 22 ± 1 °C.

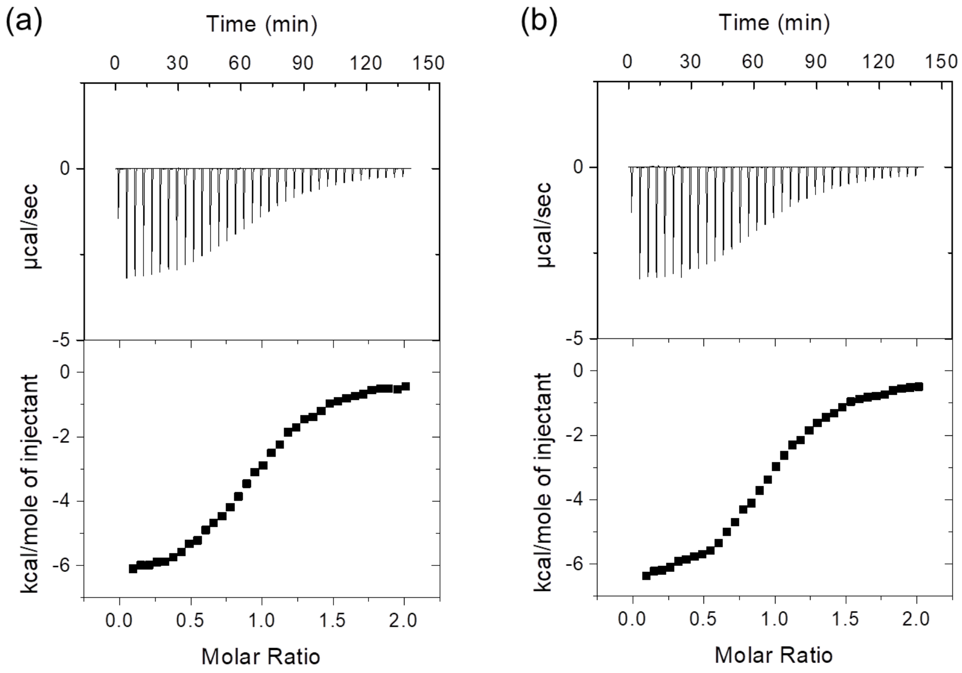

Supplementary Figure 29. (a,b) Experimental ITC measurements and binding isotherms of **6** (1.50×10^{-4} M) with me-β-D-glc (4.00×10^{-3} M) in 5% (v/v) DMSO/CH_2Cl_2 (containing 0.04–0.06% water) at 22 ± 1 °C.

Supplementary Table 8. Thermodynamic parameters between **6** and me-β-D-glc in 5% (v/v) DMSO/CH_2Cl_2 (containing 0.04–0.06% water) at 22 ± 1 °C.

	Log *K*	Δ*G*° (kJ mol^{-1})	Δ*H*° (kJ mol^{-1})	TΔ*S*° (kJ mol^{-1})
(a)	13.5	−76.2	−56.2	+20.0
(b)	13.6	−76.8	−55.7	+20.9

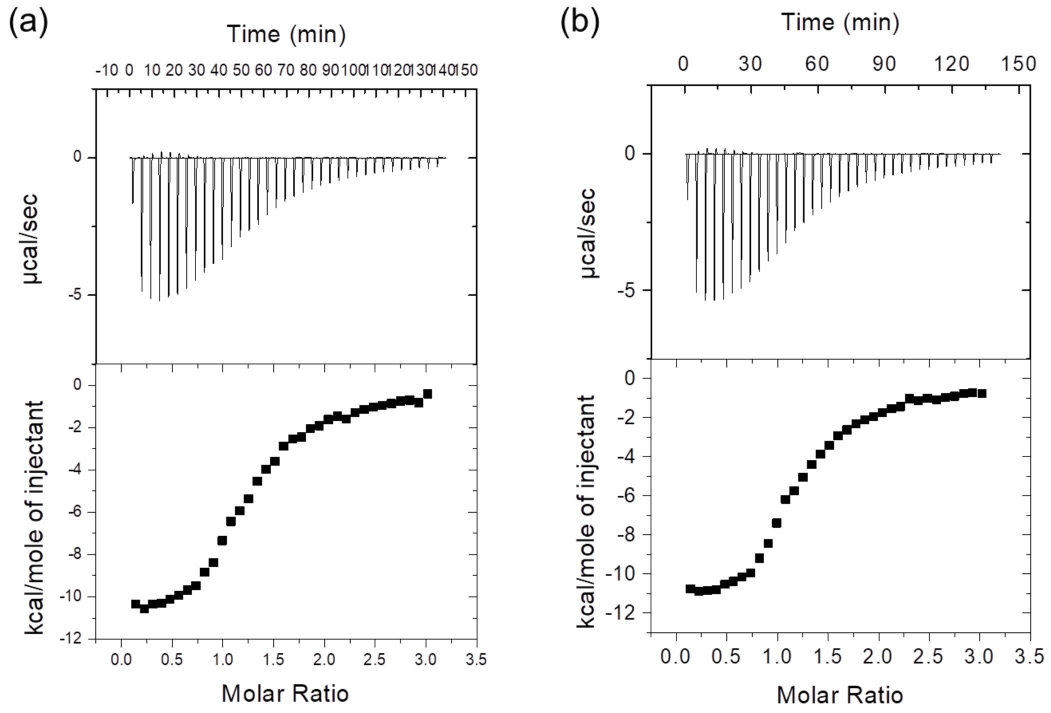

Supplementary Figure 30. (a,b) Experimental ITC measurements and binding isotherms of **6** (1.00×10^{-4} M) with me-β-D-gal (4.00×10^{-3} M) in 5% (v/v) DMSO/CH_2Cl_2 (containing 0.04–0.06% water) at 22 ± 1 °C.

Supplementary Table 9. Thermodynamic parameters between **6** and me-β-D-gal in 5% (v/v) DMSO/CH_2Cl_2 (containing 0.04–0.06% water) at 22 ± 1 °C.

	Log *K*	Δ*G*° (kJ mol^{-1})	Δ*H*° (kJ mol^{-1})	TΔ*S*° (kJ mol^{-1})
(a)	13.1	−74.1	−130.4	-56.3
(b)	13.2	−74.6	−130.1	-55.4

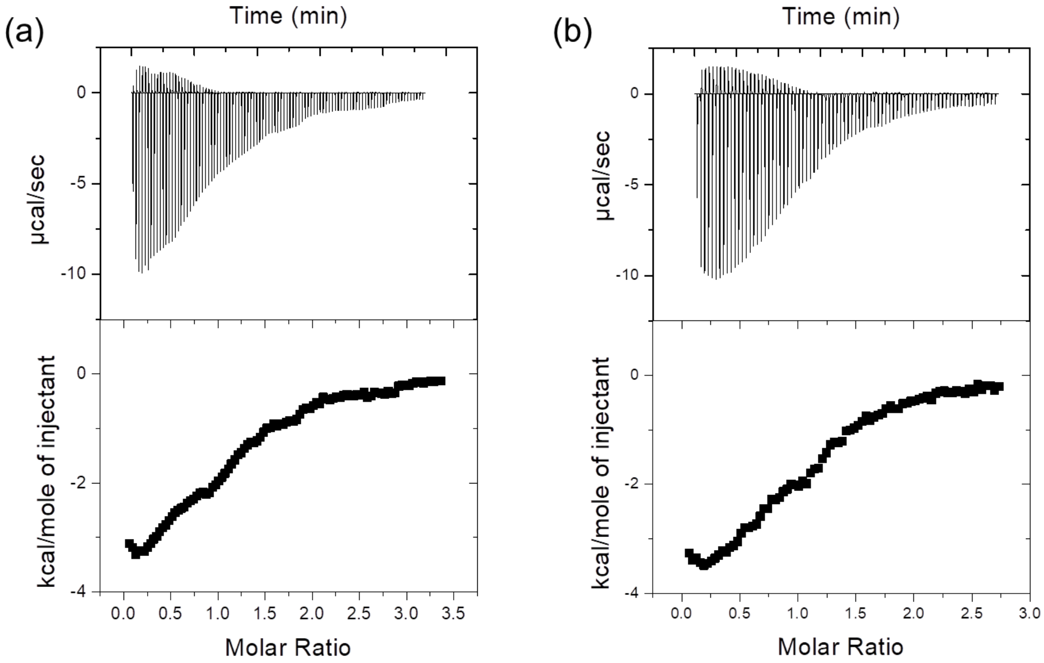

Supplementary Figure 31. (a,b) Experimental ITC measurements and binding isotherms of **6** (1.00×10^{-3} M) with methyl β-D-xylopyranoside (me-β-D-xyl) (1.50×10^{-2} M) in 5% (v/v) DMSO/CH_2Cl_2 (containing 0.04–0.06% water) at 22 ± 1 °C.

Supplementary Table 10. Thermodynamic parameters between **6** and me-β-D-xyl in 5% (v/v) DMSO/CH_2Cl_2 (containing 0.04–0.06% water) at 22 ± 1 °C.

	Log *K*	Δ*G*° (kJ mol^{-1})	Δ*H*° (kJ mol^{-1})	TΔ*S*° (kJ mol^{-1})
(a)	10.3	−58.0	−38.2	+19.8
(b)	10.0	−56.3	−39.6	+16.7

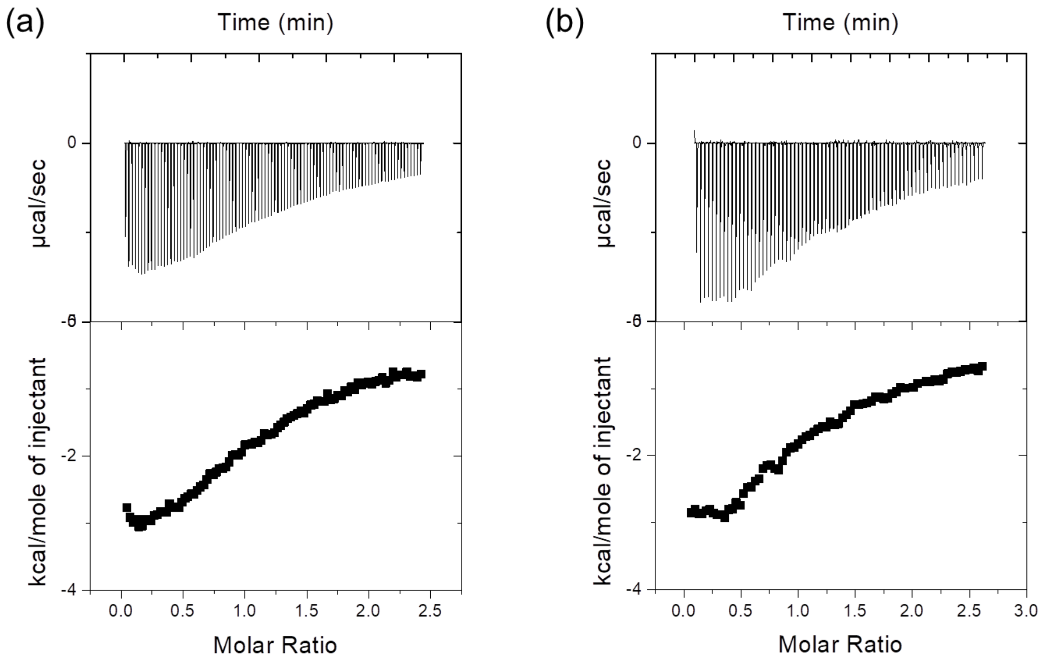

Supplementary Figure 32. (a,b) Experimental ITC measurements and binding isotherms of **6** (7.00×10^{-4} M) with me-α-D-gal (8.00×10^{-3} M) in 5% (v/v) DMSO/CH_2Cl_2 (containing 0.04–0.06% water) at 22 ± 1 °C. (injection volume: (a) 3 μL, (b) 4 μL)

Supplementary Table 11. Thermodynamic parameters between **6** and me-α-D-gal in 5% (v/v) DMSO/CH_2Cl_2 (containing 0.04–0.06% water) at 22 ± 1 °C.

	Log *K*	Δ*G*° (kJ mol^{-1})	Δ*H*° (kJ mol^{-1})	TΔ*S*° (kJ mol^{-1})
(a)	10.2	−57.5	−41.3	+16.2
(b)	10.0	−56.8	−43.1	+13.7

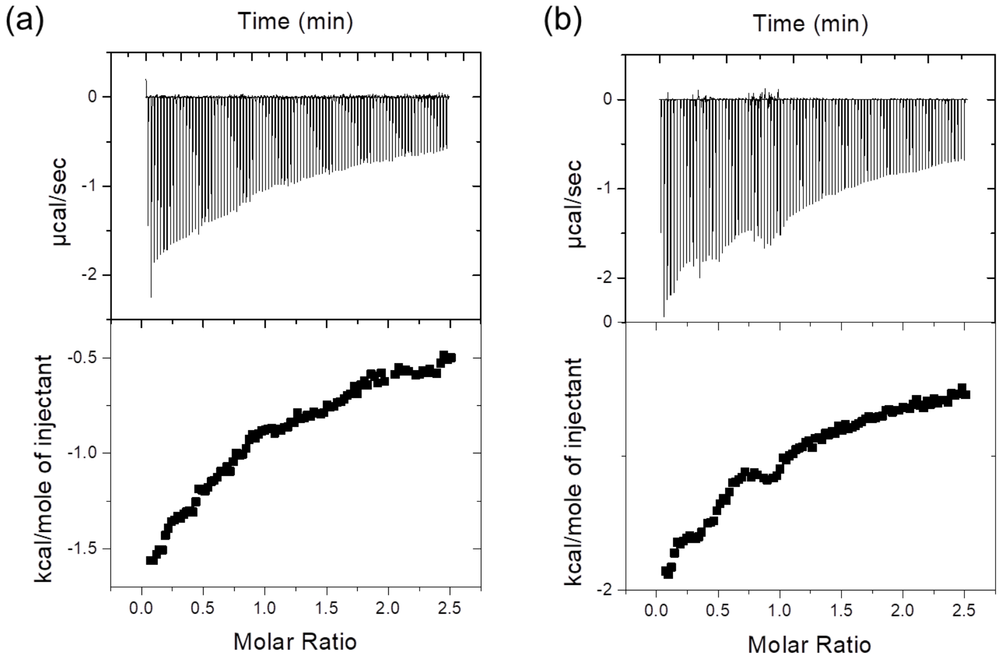

Supplementary Figure 33. (a,b) Experimental ITC measurements and binding isotherms of **6** (7.00×10^{-4} M) with methyl α-D-mannopyranoside (me-α-D-man) (8.00×10^{-3} M) in 5% (v/v) DMSO/CH_2Cl_2 (containing 0.04–0.06% water) at 22 ± 1 °C.

Supplementary Table 12. Thermodynamic parameters between **6** and me-α-D-man in 5% (v/v) DMSO/CH_2Cl_2 (containing 0.04–0.06% water) at 22 ± 1 °C.

	Log *K*	Δ*G*° (kJ mol^{-1})	Δ*H*° (kJ mol^{-1})	TΔ*S*° (kJ mol^{-1})
(a)	9.8	−55.3	−24.2	+31.1
(b)	10.1	−56.6	−25.2	+31.4

6. X-ray crystallography analyses

6.1 Crystal growing

4⊃α-D-GF·H_2O: Solid D-galactose was dissolved in a chloroform solution containing diimine **4**. Pentane was slowly introduced to this solution through vapor diffusion for a few days at room temperature to yield single crystals suitable for the X-ray diffraction.

(**6**-*MP*)$_2$⊃(me-β-D-glc)$_2$: Complex between **6** and me-β-D-glc, obtained from a reaction mixture, was dissolved in 1,2-dichloroethane in a test tube. To this solution, pentane was slowly vapor-diffused for a few days at room temperature to yield single crystals suitable for the X-ray diffraction.

(**6**-*MM*)$_2$⊃(me-β-D-gal·2H_2O)$_2$ · (**6**-*PP*)$_2$⊃(me-β-L-gal·2H_2O)$_2$: Racemic crystallization was conducted to obtain single crystals suitable for the X-ray diffraction. To a dichloromethane/heptane (3:1) solution containing 1:1 ratio of complex **6**/me-β-D-gal and **6**/me-β-L-gal[4], pentane was slowly vapor-diffused for a few days at room temperature to yield single crystals suitable for the X-ray diffraction.

6.2 Summary

4⊃α-D-GF·H_2O: Crystal Data for $C_{177}H_{163}Cl_3N_{18}O_9$ (M =2792.59 g/mol): monoclinic, space group $P2_1$ (no. 4), a = 23.3393(8) Å, b = 15.6893(5) Å, c = 23.6427(8) Å, β = 105.5805(19)°, V = 8339.3(5) Å^3, Z = 2, T = 296.15 K, μ(CuKα) = 0.973 mm^{-1}, *Dcalc* = 1.112 g/cm^3, 42927 reflections measured (3.93° ≤ 2Θ ≤ 115.996°), 17520 unique (R_{int} = 0.1018, R_{sigma} = 0.1389) which were used in all calculations. The final R_1 was 0.1013 (I > 2σ(I)) and wR_2 was 0.3440 (all data).

(**6**-*MP*)$_2$⊃(me-β-D-glc)$_2$: Crystal Data for $C_{190}H_{166}ClN_{18}O_8$ (M =2864.85 g/mol): monoclinic, space group $P2_1$ (no. 4), a = 25.586(5) Å, b = 20.521(4) Å, c = 37.912(8) Å, β = 97.57(3)°, V = 19732(7) Å^3, Z = 4, T = 100 K, μ(synchrotron) = 0.070 mm^{-1}, *Dcalc* = 0.964 g/cm^3, 159631 reflections measured (2.226° ≤ 2Θ ≤ 57.998°), 90701 unique (R_{int} = 0.1818, R_{sigma} = 0.4600) which were used in all calculations. The final R_1 was 0.0677 (I > 2σ(I)) and wR_2 was 0.2007 (all data).

(**6**-*MM*)$_2$⊃(me-β-D-gal·2H_2O)$_2$ · (**6**-*PP*)$_2$⊃(me-β-L-gal·2H_2O)$_2$: Crystal Data for $C_{187}H_{177}ClN_{18}O_{10}$ (M =2871.91 g/mol): monoclinic, space group $P112_1$/n (no. 14), a = 19.029(4) Å, b = 30.708(6) Å, c = 71.364(14) Å, β = 89.84(3)°, V = 41701(14) Å^3, Z = 8, T = 100 K, μ(synchrotron) = 0.067 mm^{-1}, *Dcalc* = 0.915 g/cm^3, 398998 reflections measured (3.76° ≤ 2Θ ≤ 57°), 110278 unique (R_{int} = 0.2027, R_{sigma} = 0.2066) which were used in all calculations. The final R_1 was 0.1127 (I > 2σ(I)) and wR_2 was 0.3553 (all data).

CIF validation of **4**⊃α-D-GF·H_2O, (**6**-*MP*)$_2$⊃(me-β-D-glc)$_2$, and (**6**-*MM*)$_2$⊃(me-β-D-gal·2H_2O)$_2$ · (**6**-*PP*)$_2$⊃(me-β-L-gal·2H_2O)$_2$

A- and B- level alerts were founded by the checkCIF routine from IUCr, all of which were attributed to weak crystal diffraction, highly disordered solvent molecules, data incompleteness, and moderate resolution. The alerts are listed below

CheckCIF for **4**⊃α-D-GF·H_2O gives 10 B level alerts, all attributed to the weak data quality mentioned above.

THETM01_ALERT_3_B The value of sine(theta_max)/wavelength is less than 0.575
Calculated sin(theta_max)/wavelength = 0.5500
PLAT029_ALERT_3_B _diffrn_measured_fraction_theta_full value Low . 0.955 Why?
PLAT234_ALERT_4_B Large Hirshfeld Difference N6 --C7 . 0.28 Ang.
PLAT234_ALERT_4_B Large Hirshfeld Difference C11 --C12 . 0.26 Ang.
PLAT234_ALERT_4_B Large Hirshfeld Difference C16 --C17 . 0.26 Ang.
PLAT340_ALERT_3_B Low Bond Precision on C-C Bonds .... 0.02935 Ang.
PLAT412_ALERT_2_B Short Intra XH3 .. XHn H17M ..H60 . 1.79 Ang. x,y,z = 1_555 Check
PLAT414_ALERT_2_B Short Intra D-H..H-X H6AA ..H5A . 1.88 Ang. x,y,z = 1_555 Check
PLAT417_ALERT_2_B Short Inter D-H..H-D H1A ..H132 . 2.04 Ang. x,y,z = 1_555 Check
PLAT420_ALERT_2_B D-H Bond Without Acceptor O5A --H5A . Please Check

CheckCIF for (**6**-*MP*)$_2$⊃(me-β-D-glc)$_2$ gives 2 A and 17 B level alerts, all attributed to the weak data quality mentioned above.

PLAT026_ALERT_3_A Ratio Observed / Unique Reflections (too) Low .. 17% Check
PLAT029_ALERT_3_A _diffrn_measured_fraction_theta_full value Low . 0.917 Why?
RINTA01_ALERT_3_B The value of Rint is greater than 0.18 Rint given 0.182
PLAT242_ALERT_2_B Low 'MainMol' Ueq as Compared to Neighbors of C186 Check
PLAT242_ALERT_2_B Low 'MainMol' Ueq as Compared to Neighbors of C363 Check
PLAT242_ALERT_2_B Low 'MainMol' Ueq as Compared to Neighbors of C379 Check
PLAT315_ALERT_2_B Singly Bonded Carbon Detected (H-atoms Missing). C399 Check
PLAT315_ALERT_2_B Singly Bonded Carbon Detected (H-atoms Missing). C403 Check
PLAT315_ALERT_2_B Singly Bonded Carbon Detected (H-atoms Missing). C404 Check
PLAT315_ALERT_2_B Singly Bonded Carbon Detected (H-atoms Missing). C408 Check
PLAT340_ALERT_3_B Low Bond Precision on C-C Bonds .... 0.01773 Ang.

PLAT369_ALERT_2_B Long C(sp2)-C(sp2) Bond C41 - C60 . 1.58 Ang.

PLAT369_ALERT_2_B Long C(sp2)-C(sp2) Bond C62 - C77 . 1.57 Ang.

PLAT369_ALERT_2_B Long C(sp2)-C(sp2) Bond C117 - C118 . 1.57 Ang.

PLAT369_ALERT_2_B Long C(sp2)-C(sp2) Bond C235 - C236 . 1.59 Ang.

PLAT369_ALERT_2_B Long C(sp2)-C(sp2) Bond C241 - C253 . 1.60 Ang.

PLAT372_ALERT_2_B Short C(sp)-C(sp) Bond C152 - C153 . 1.00 Ang.

PLAT412_ALERT_2_B Short Intra XH3 .. XHn H11 ..H17M . 1.73 Ang. x,y,z = 1_555 Check

PLAT412_ALERT_2_B Short Intra XH3 .. XHn H19M ..H71 . 1.79 Ang. x,y,z = 1_555 Check

CheckCIF for (**6**-*MM*)$_2$⊃(me-β-D-gal·2H_2O)$_2$ · (**6**-*PP*)$_2$⊃(me-β-L-gal·2H_2O)$_2$ gives 1 A and 11 B level alerts, all attributed to the weak data quality mentioned above.

PLAT026_ALERT_3_A Ratio Observed / Unique Reflections (too) Low .. 25% Check

RINTA01_ALERT_3_B The value of Rint is greater than 0.18 Rint given 0.203

PLAT020_ALERT_3_B The Value of Rint is Greater Than 0.12 .... 0.203 Report

PLAT084_ALERT_3_B High wR2 Value (i.e. > 0.25) .... 0.36 Report

PLAT242_ALERT_2_B Low 'MainMol' Ueq as Compared to Neighbors of C170 Check

PLAT242_ALERT_2_B Low 'MainMol' Ueq as Compared to Neighbors of C391 Check

PLAT260_ALERT_2_B Large Average Ueq of Residue Including C142 0.306 Check

PLAT369_ALERT_2_B Long C(sp2)-C(sp2) Bond C76 - C77 . 1.57 Ang.

PLAT369_ALERT_2_B Long C(sp2)-C(sp2) Bond C95 - C101 . 1.60 Ang.

PLAT369_ALERT_2_B Long C(sp2)-C(sp2) Bond C117 - C118 . 1.60 Ang.

PLAT369_ALERT_2_B Long C(sp2)-C(sp2) Bond C292 - C298 . 1.60 Ang.

PLAT369_ALERT_2_B Long C(sp2)-C(sp2) Bond C314 - C315 . 1.59 Ang.

Supplementary Table 13. Crystal data and structure refinement for **4**⊃α-D-GF·H_2O.

Identification code	**4**⊃α-D-GF·H_2O
Empirical formula	$C_{177}H_{163}Cl_3N_{18}O_9$
Formula weight	2792.59
Temperature/K	296
Crystal system	monoclinic
Space group	$P2_1$
a/Å	23.3393(8)
b/Å	15.6893(5)
c/Å	23.6427(8)
α/°	90
β/°	105.5805(19)
γ/°	90
Volume/Å^3	8339.3(5)
Z	2
ρ_{calc}g/cm^3	1.112
μ/mm^{-1}	0.973
F(000)	2948.0
Crystal size/mm^3	0.213 × 0.125 × 0.065
Radiation	CuKα (λ = 1.54184)
2Θ range for data collection/°	3.93 to 115.996
Index ranges	-25 ≤ h ≤ 21, -17 ≤ k ≤ 12, -26 ≤ l ≤ 23
Reflections collected	42927
Independent reflections	17520 [R_{int} = 0.1018, R_{sigma} = 0.1389]
Data/restraints/parameters	17520/1062/1903
Goodness-of-fit on F^2	0.984
Final R indexes [I>=2σ (I)]	R_1 = 0.1013, wR_2 = 0.2531
Final R indexes [all data]	R_1 = 0.2008, wR_2 = 0.3440
Largest diff. peak/hole / e Å^{-3}	0.34/-0.39
Flack parameter	0.21(2)
CCDC number	2287025

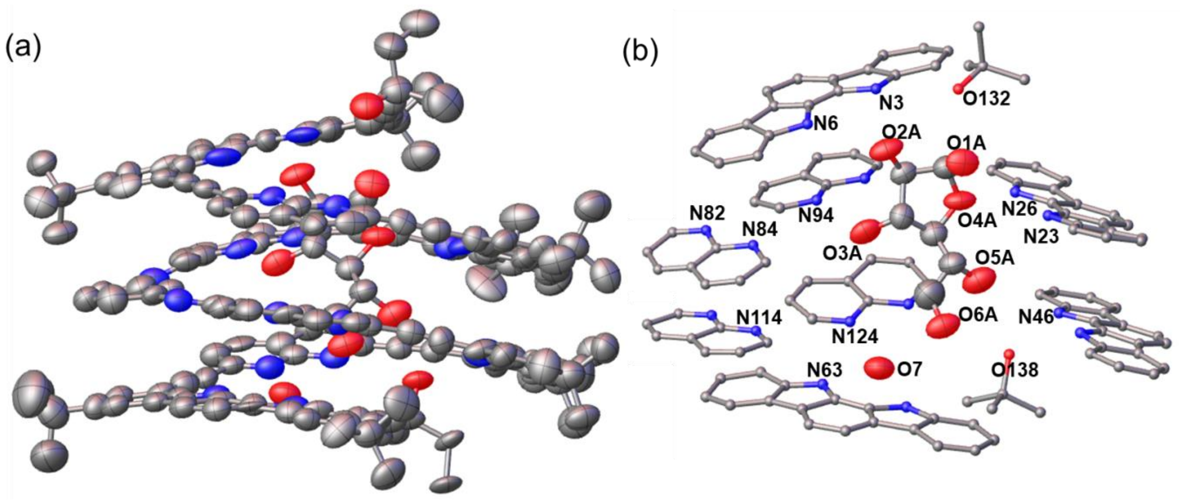

Supplementary Figure 34. (a,b) Thermal ellipsoid representations of X-ray crystal structure of **4**⊃α-D-GF·H_2O. In (b), CH hydrogens, *t*-butyls and ethynyl bonds are omitted for clarity.

Supplementary Table 14. Hydrogen bonding distances (Å) in the crystal structure of **4**⊃α-D-GF·H_2O.

Donor···Acceptor	Distance (Å)
O1A···N84	2.74
O132···O1A	2.70
O2A···N82	2.91
N6···O2A	3.03
N3···O2A	3.22
O3A···N94	2.87
N23···O4A	3.22
N26···O4A	3.07
N46···O5A	3.27
O6A···N114	2.94
O138···O6A	2.81
O7···O6A	2.87
N63···O7	2.86
O7···N124	2.96

Supplementary Table 15. Crystal data and structure refinement for (**6**-*MP*)$_2$⊃(me-β-D-glc)$_2$.

Identification code	(**6**-*MP*)$_2$⊃(me-β-D-glc)$_2$
Empirical formula	$C_{190}H_{166}ClN_{18}O_8$
Formula weight	2864.85
Temperature/K	100
Crystal system	monoclinic
Space group	$P2_1$
a/Å	25.586(5)
b/Å	20.521(4)
c/Å	37.912(8)
α/°	90
β/°	97.57(3)
γ/°	90
Volume/Å^3	19732(7)
Z	4
ρ_{calc}g/cm^3	0.964
μ/mm^{-1}	0.070
F(000)	6052.0
Crystal size/mm^3	0.1 × 0.04 × 0.02
Radiation	synchrotron (λ = 0.700000)
2Θ range for data collection/°	2.226 to 57.998
Index ranges	-35 ≤ h ≤ 35, -25 ≤ k ≤ 25, -52 ≤ l ≤ 52
Reflections collected	159631
Independent reflections	90701 [R_{int} = 0.1818, R_{sigma} = 0.4600]
Data/restraints/parameters	90701/1418/3812
Goodness-of-fit on F^2	0.633
Final R indexes [I>=2σ (I)]	R_1 = 0.0677, wR_2 = 0.1456
Final R indexes [all data]	R_1 = 0.3024, wR_2 = 0.2007
Largest diff. peak/hole / e Å^{-3}	0.33/-0.22
Flack parameter	0.30(10)
CCDC number	2287027

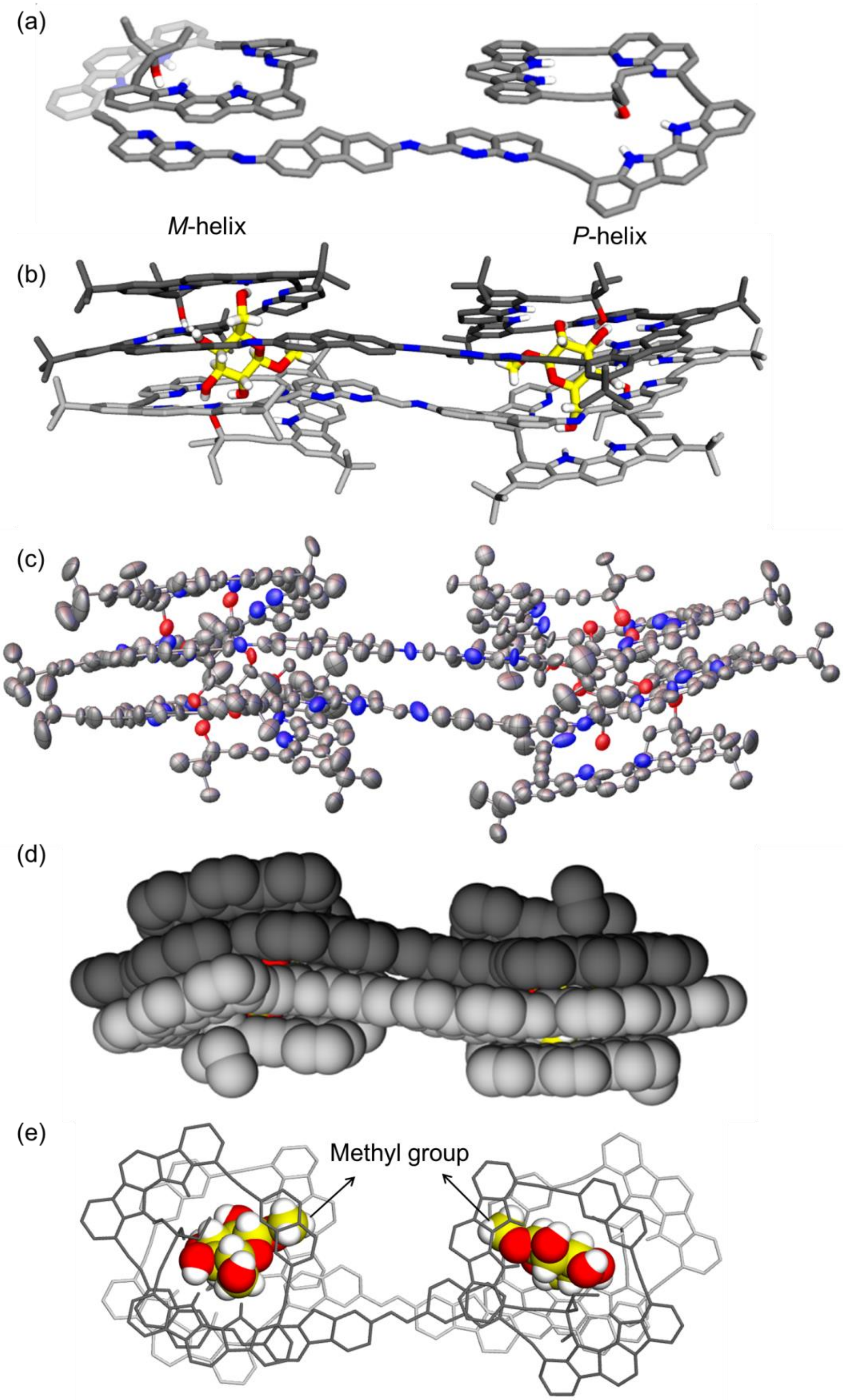

Supplementary Figure 35. Tube representations of (a) a monomeric strand of **6** and (b) the (**6**-*MP*)$_2$⊃(me-β-D-glc)$_2$ complex in the X-ray crystal structure. (c) Thermal ellipsoid and (d) Space-filling representations of the X-ray crystal structure of (**6**-*MP*)$_2$⊃(me-β-D-glc)$_2$. (e) Top view of the X-ray crystal structure of (**6**-*MP*)$_2$⊃(me-β-D-glc)$_2$. The methyl groups of me-β-D-glc are positioned between the aryl planes of two different strands.

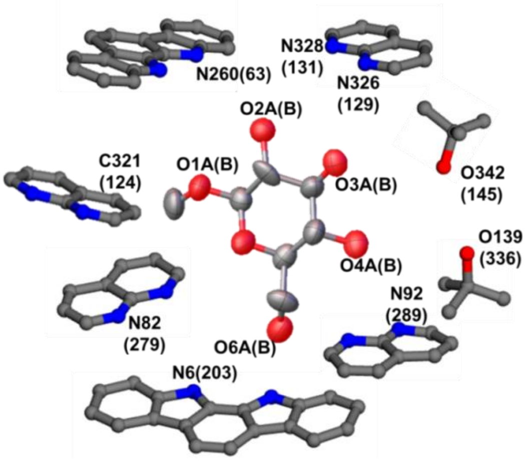

Supplementary Figure 36. Ellipsoid representation of (**6**-*MP*)$_2$⊃(me-β-ᴅ-glc)$_2$. CH hydrogens, *t*-butyls and ethynyl bonds are omitted for clarity.

Supplementary Table 16. Hydrogen bonding distances (Å) in the crystal structure of (**6**-*MP*)$_2$⊃(me-β-ᴅ-glc)$_2$.

Donor···Acceptor	Distance (Å)	Donor···Acceptor	Distance (Å)
C321···O1A	3.13	C124···O1B	3.20
O2A···N328	2.89	O2B···N131	2.94
N260···O2A	3.03	N63···O2B	2.97
O3A···N326	2.68	O3B···N129	2.72
O342···O3A	2.74	O145···O3B	2.73
O139···O4A	2.76	O336···O4B	2.76
O4A···N92	2.80	O4B···N289	2.73
N6···O6A	2.89	N203···O6B	2.82
O6A···N82	2.87	O6B···N279	2.87

Supplementary Table 17. Crystal data and structure refinement for (**6**-*MM*)$_2$⊃(me-β-D-gal·2H_2O)$_2$ · (**6**-*PP*)$_2$⊃(me-β-L-gal·2H_2O)$_2$.

Identification code	(**6**-*MM*)$_2$⊃(me-β-D-gal·2H_2O)$_2$ · (**6**-*PP*)$_2$⊃(me-β-L-gal·2H_2O)$_2$
Empirical formula	$C_{187}H_{177}ClN_{18}O_{10}$
Formula weight	2871.91
Temperature/K	100
Crystal system	monoclinic
Space group	$P112_1/n$
a/Å	19.029(4)
b/Å	30.708(6)
c/Å	71.364(14)
α/°	90
β/°	89.84(3)
γ/°	90
Volume/Å^3	41701(14)
Z	8
ρ_{calc}g/cm^3	0.915
μ/mm^{-1}	0.067
F(000)	12176.0
Crystal size/mm^3	0.205 × 0.2 × 0.17
Radiation	synchrotron (λ = 0.700000)
2Θ range for data collection/°	3.76 to 57
Index ranges	$-25 \le h \le 25$, $-41 \le k \le 41$, $-97 \le l \le 97$
Reflections collected	398998
Independent reflections	110278 [R_{int} = 0.2027, R_{sigma} = 0.2066]
Data/restraints/parameters	110278/716/4093
Goodness-of-fit on F^2	0.964
Final R indexes [I>=2σ (I)]	R_1 = 0.1127, wR_2 = 0.2994
Final R indexes [all data]	R_1 = 0.2514, wR_2 = 0.3553
Largest diff. peak/hole / e Å^{-3}	0.77/-0.58
CCDC number	2287026

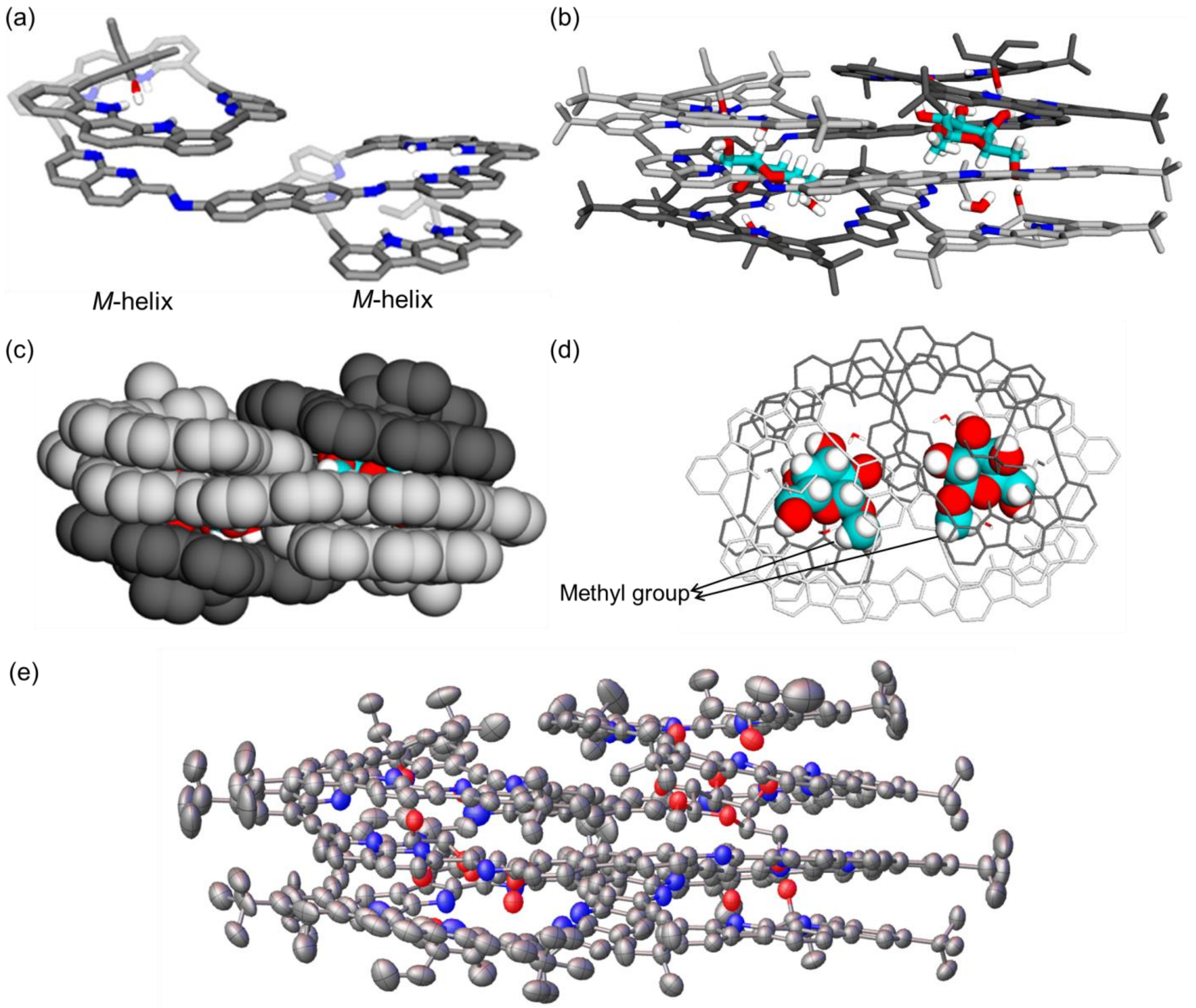

Supplementary Figure 37. Tube representations of (a) a monomeric strand of **6** and (b) the (**6**-*MM*)$_2$⊃(me-β-D-gal·$2H_2O$)$_2$ complex in the X-ray crystal structure. (c) Space-filling representation of the X-ray crystal structure of (**6**-*MM*)$_2$⊃(me-β-D-gal·$2H_2O$)$_2$. (d) Top view of the X-ray crystal structure of (**6**-*MM*)$_2$⊃(me-β-D-gal·$2H_2O$)$_2$. The methyl groups of me-β-D-gal are shielded by aryl planes. (e) Thermal ellipsoid representation of the X-ray crystal structure of (**6**-*MM*)$_2$⊃(me-β-D-gal·$2H_2O$)$_2$.

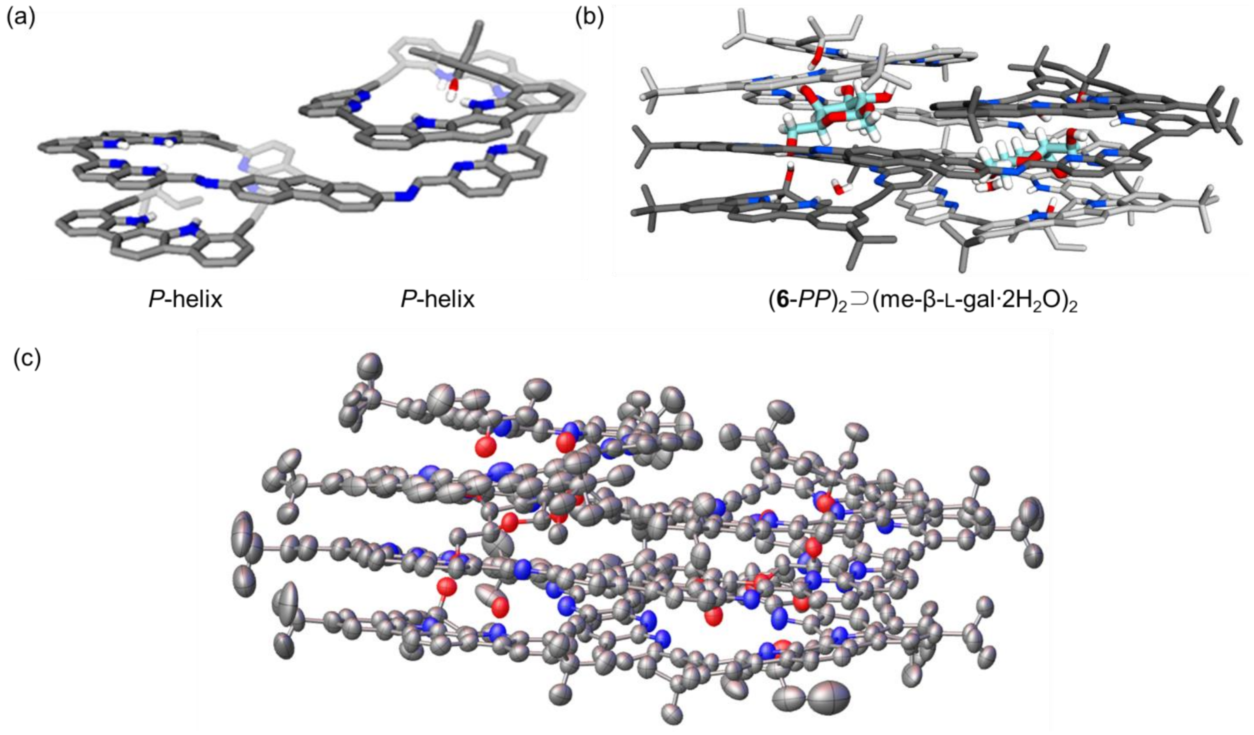

Supplementary Figure 38. Tube representations of (a) a monomeric strand of **6** and (b) the (**6**-*PP*)$_2$⊃(me-β-L-gal·2H_2O)$_2$ complex in the X-ray crystal structure. (c) Thermal ellipsoid representation of the X-ray crystal structure of (**6**-*PP*)$_2$⊃(me-β-L-gal·2H_2O)$_2$.

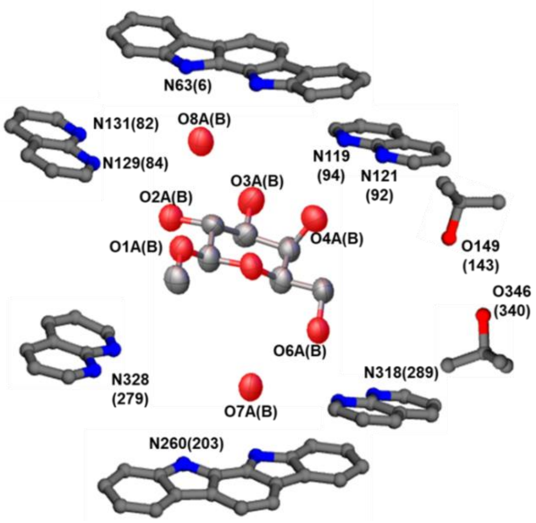

Supplementary Figure 39. Ellipsoid representation of (**6**-*MM*)$_2$⊃(me-β-D-gal·2H_2O)$_2$. CH hydrogens, *t*-butyls and ethynyl bonds are omitted for clarity.

Supplementary Table 18. Hydrogen bonding distances (Å) in the crystal structure of (**6**-*MM*)$_2$⊃(me-β-D-gal · 2H_2O)$_2$.

Donor···Acceptor	Distance (Å)	Donor···Acceptor	Distance (Å)
O2A···N129	2.79	O2B···N84	2.84
O3A···N119	3.08	O3B···N94	2.98
O8A···O3A	2.71	O8B···O3B	2.72
O4A···N121	2.85	O4B···N92	2.88
O149···O4A	2.50	O143···O4B	2.56
O6A···N318	2.79	O6B···N289	2.87
O346···O6A	2.60	O340···O6B	2.63
O7A···O6A	2.98	O7B···O6B	2.94
O7A···N328	3.14	O7B···N279	3.18
N260···O7A	2.85	N203···O7B	2.85
O8A···N131	3.11	O8B···N82	3.07
N63···O8A	2.80	N6···O8B	2.78

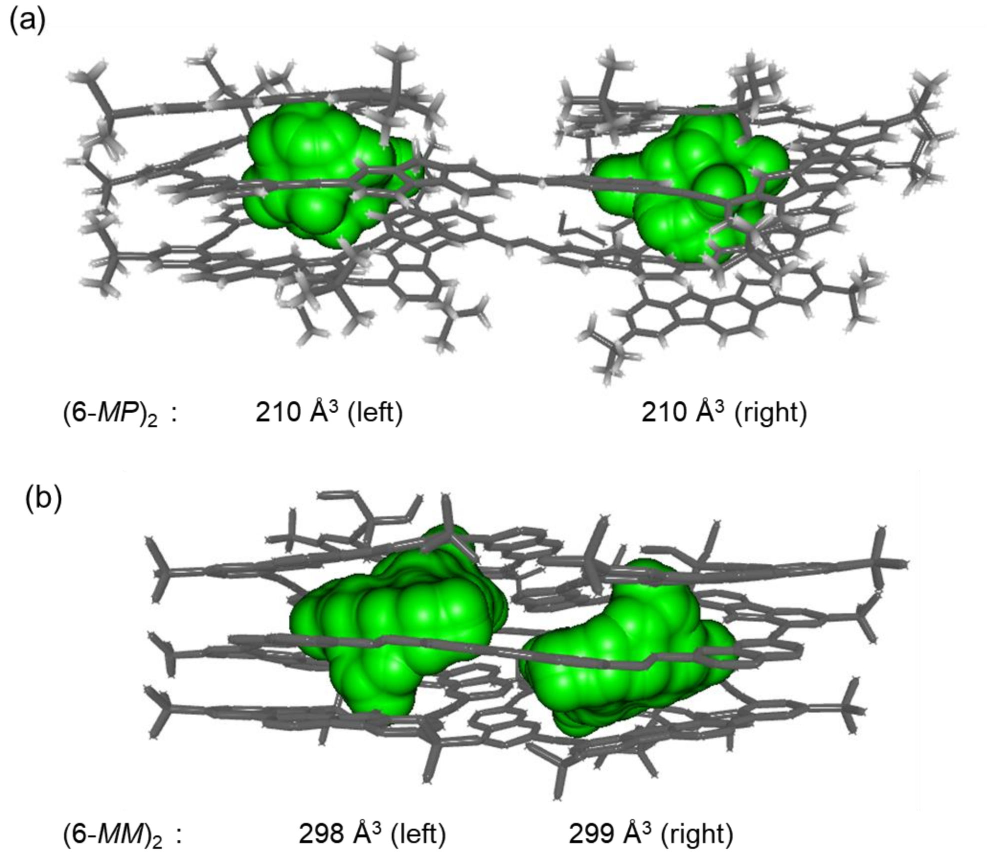

Supplementary Figure 40. Estimated cavity volumes of (a) (**6**-*MM*)$_2$ and (b) (**6**-*MP*)$_2$. For estimation, (me-β-D-gal·$2H_2O$)$_2$ and (me-β-D-glc)$_2$ were removed from the crystal structures. The cavity volumes are calculated by rolling a probe sphere of 1.4 Å inside the helical cavities using the CAVER analyst 2 software package[5].

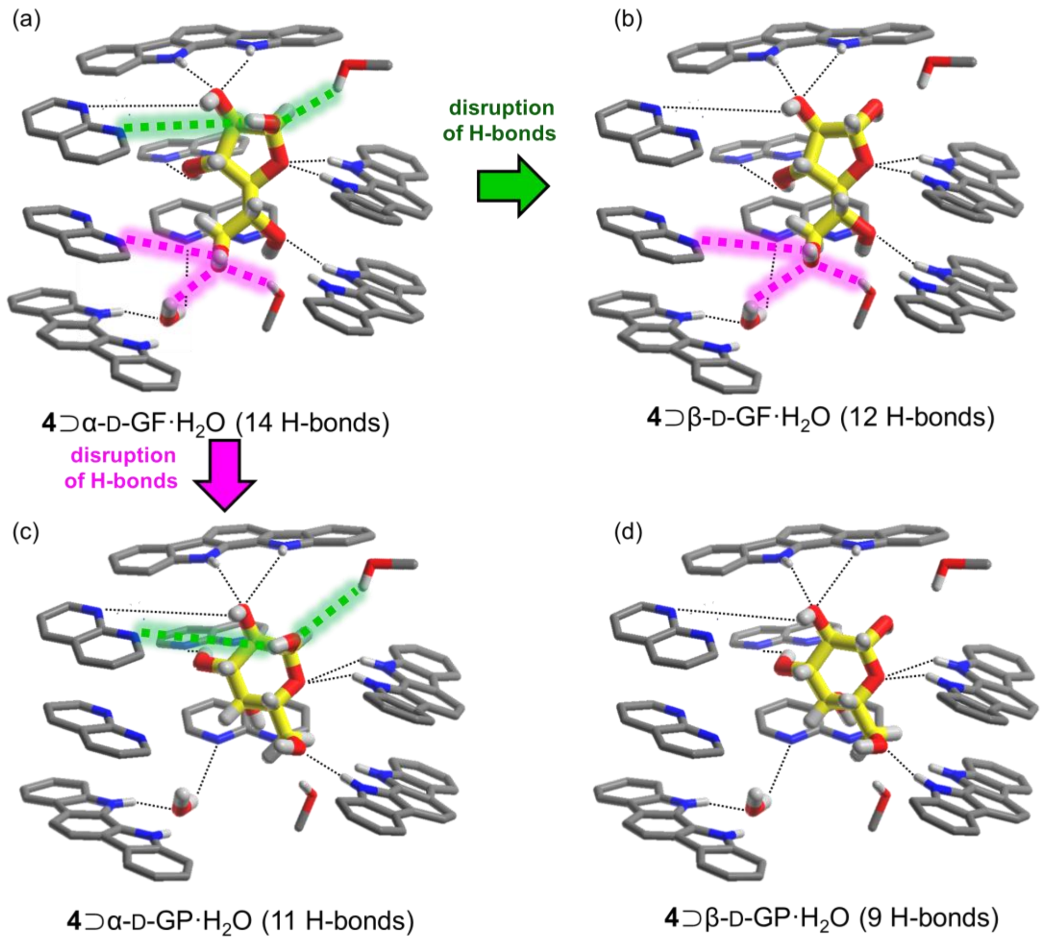

Supplementary Figure 41. Tube representations of (a) the X-ray crystal structure **4**⊃α-D-GF·H_2O, and (b-d) energy minimized molecular models depicting diimine **4** complexes with three other isomers of D-galactose: (b) β-D-GF, (c) α-D-GP, and (d) β-D-GP, based on the crystal structure. The purple- and green-colored dashed lines represent disrupted hydrogen bonds upon isomerization.

7. ^{1}H, ^{13}C NMR, and mass spectra of new compounds

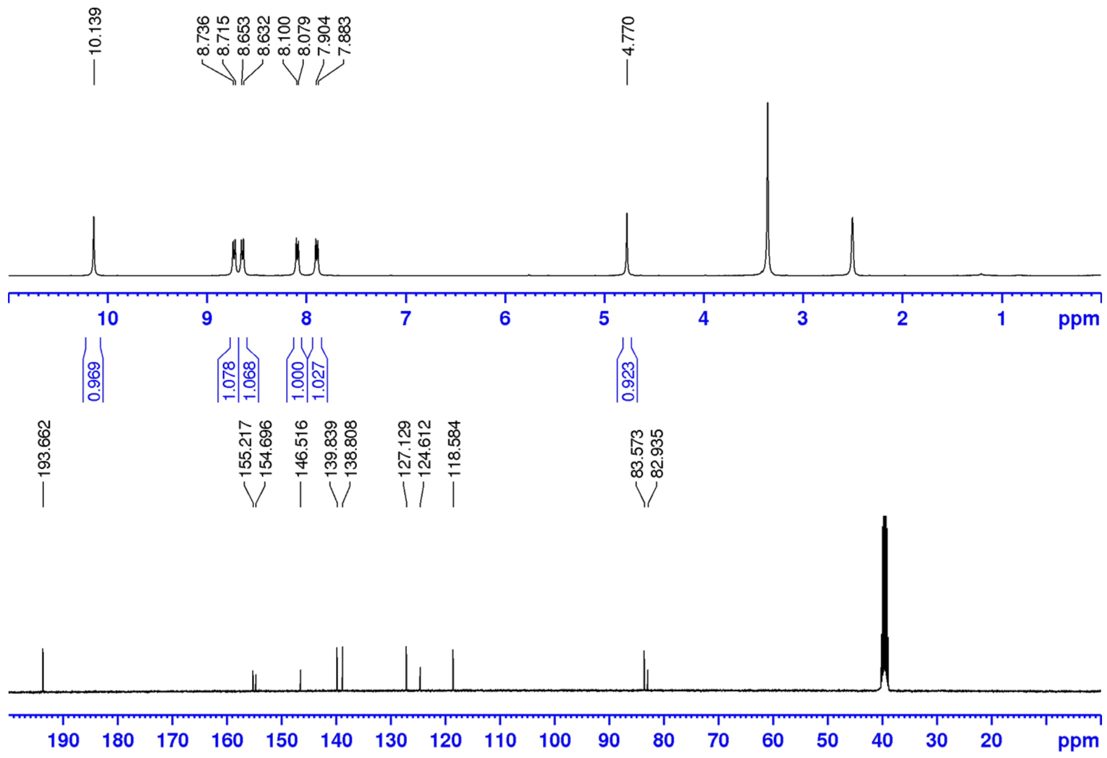

Supplementary Figure 42. ^{1}H and ^{13}C spectra of compound **S2** (DMSO-d_6, 25°C).

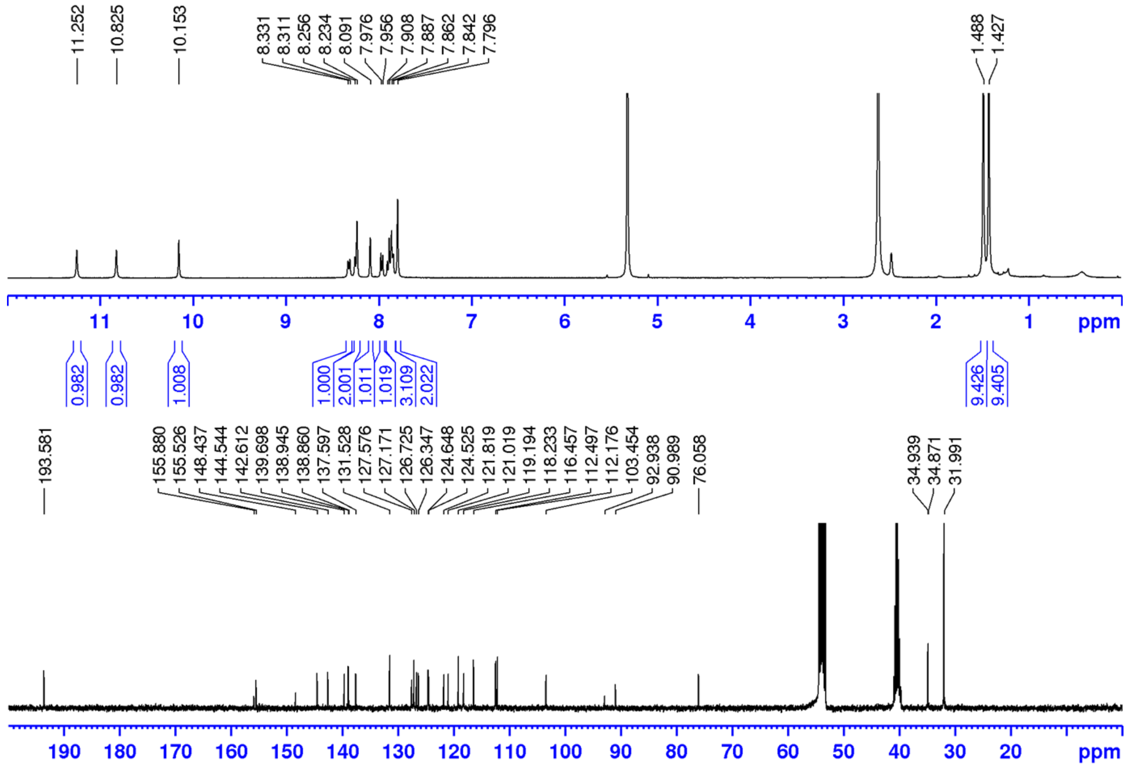

Supplementary Figure 43. ^{1}H and ^{13}C spectra of compound **S4** (10% (v/v) DMSO-d_6/CD_2Cl_2, 25°C).

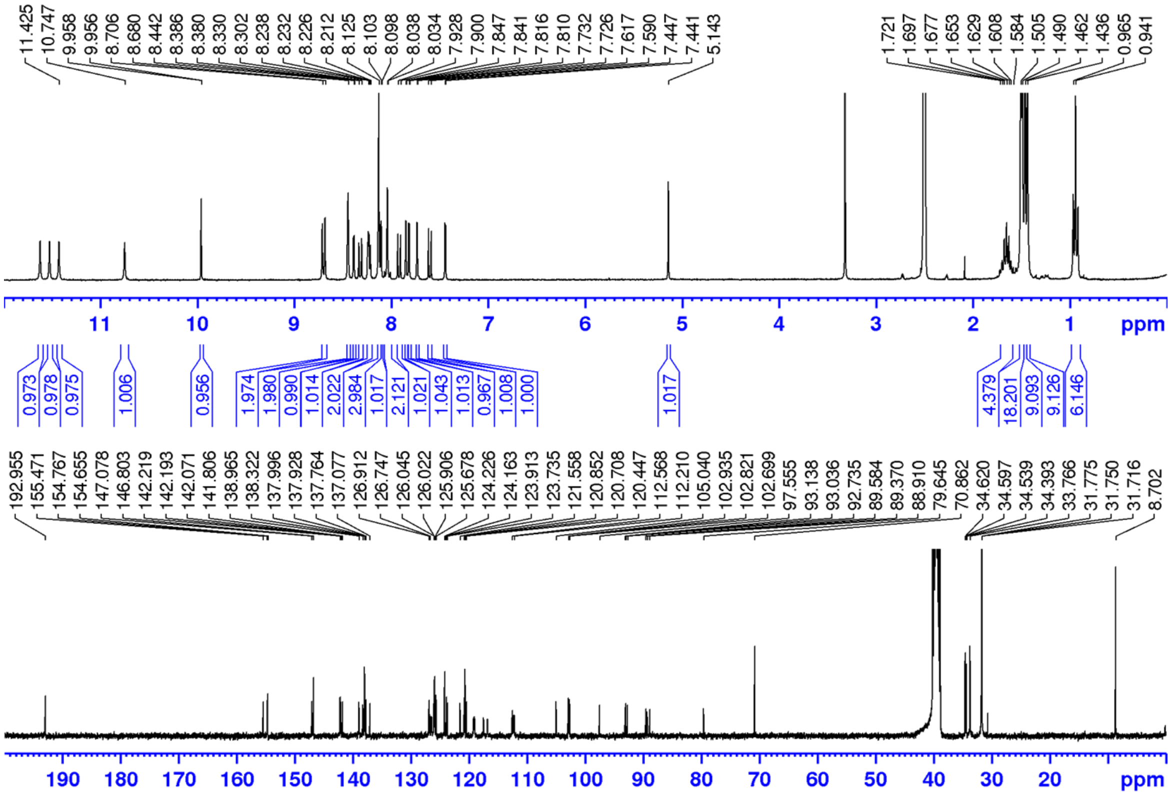

Supplementary Figure 44. ^{1}H and ^{13}C spectra of compound **1** (DMSO-d_6, 25°C)

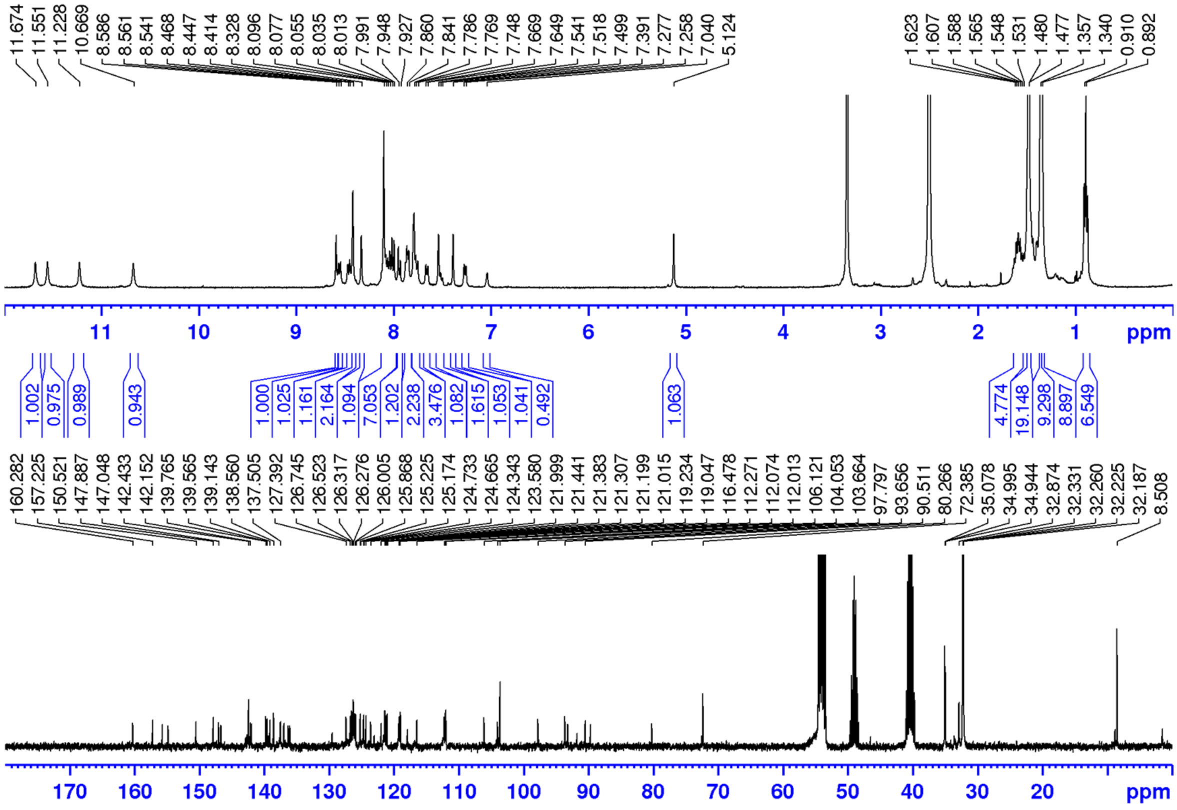

Supplementary Figure 45. ^{1}H and ^{13}C spectra of compound **4** (DMSO-d_6, 25°C).

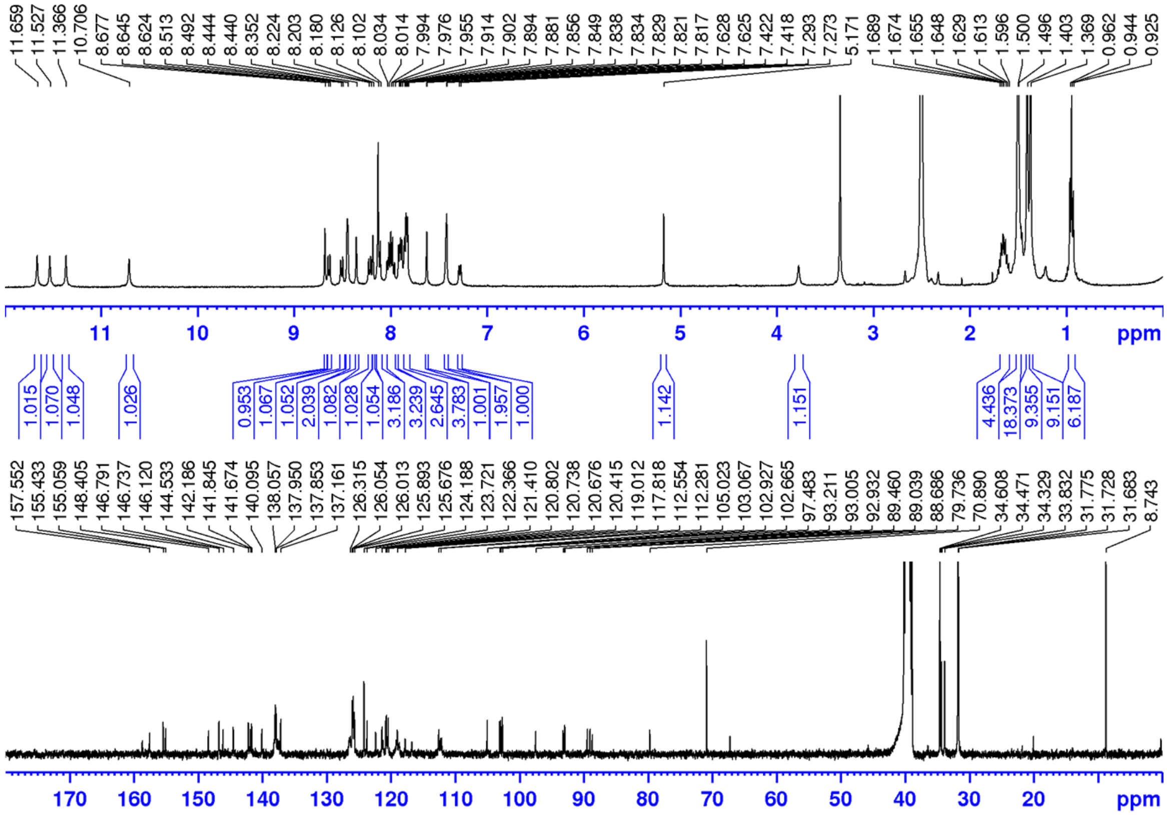

Supplementary Figure 46. ^{1}H and ^{13}C spectra of compound **6** (DMSO-d_6, 25°C)

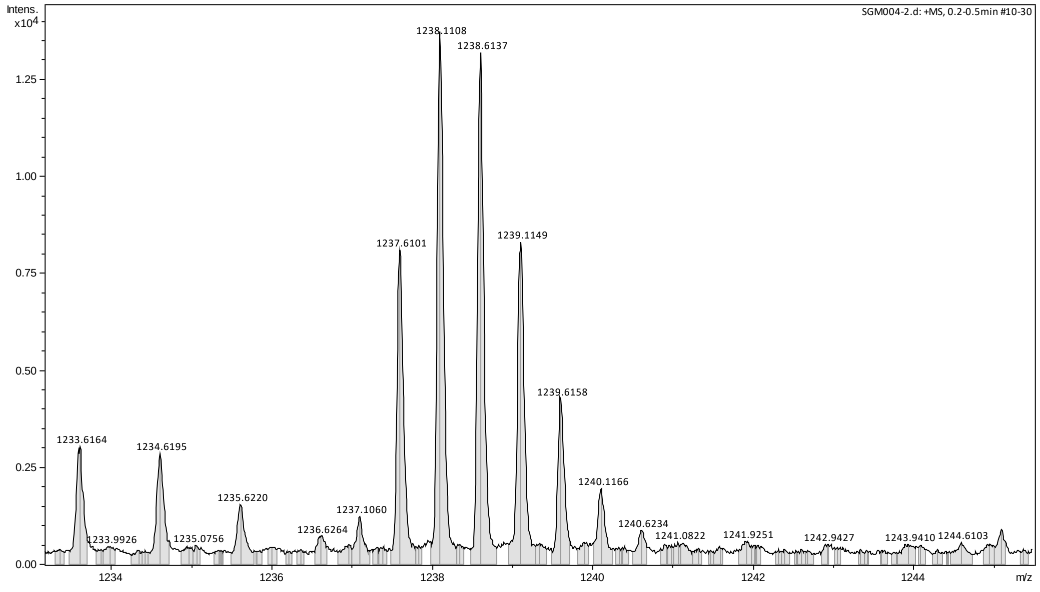

Supplementary Figure 47. ESI-HRMS spectrum of compound **4**.

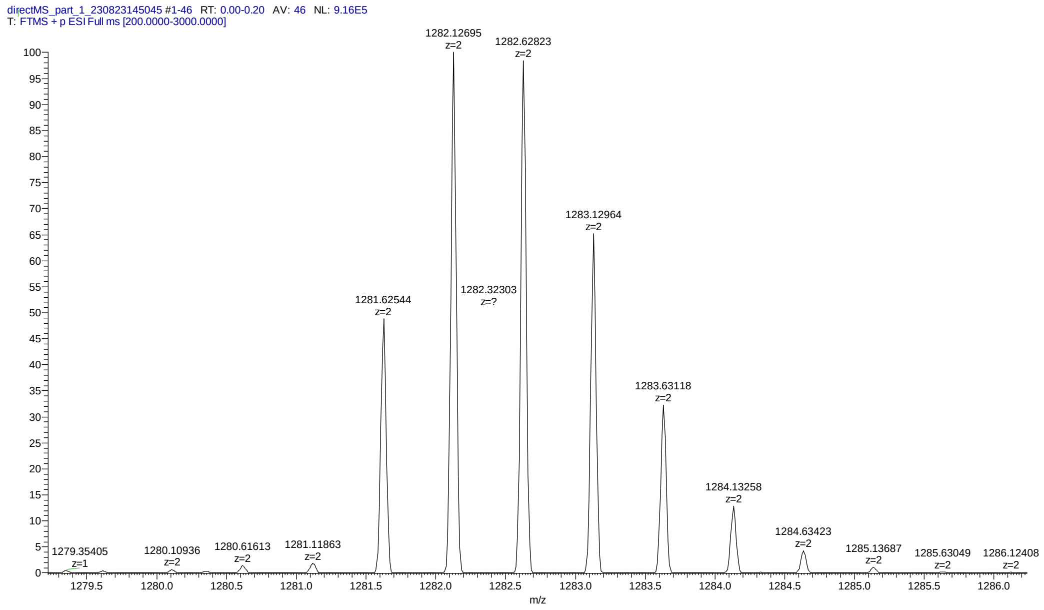

Supplementary Figure 48. Q-Exactive Plus Orbitrap MS spectrum of compound **6**.

8. Supplementary references

1. Rosin, R. Seichter, W., Schwarzer, A. & Mazik, M. 1,8-Naphthyridinecarbaldehyde and Their Methyl-Substituted Precursors: Synthesis, Molecular Structures, Supramolecular Motifs and Trapped Water Clusters. *Eur. J. Org. Chem.* 6038–6051 (2017).

2. Chang, K.-J., Moon, D., Lah, M. S. & Jeong, K.-S. Indole-Based Macrocycles as a Class of Receptors for Anions. *Angew. Chem. Int. Ed.* **44**, 7926–7929 (2005).

3. Kim, K. M., Song, G., Lee, S., Jeon, H.-G., Chae, W. & Jeong, K.-S. Template-Directed Quantitative One-Pot Synthesis of Homochiral Helical Receptors Enabling Enantioselective Binding. *Angew. Chem. Int. Ed.* **59**, 22475–22479 (2020).

4. Van der Vorm, S., Hansen, T., Overkleeft, H. S., van der Marel, G. A. & Codée, J. D. C. The influence of acceptor nucleophilicity on the glycosylation reaction mechanism. *Chem. Sci.* **8**, 1867–1875 (2017).

5. Jurcik, A. et al. CAVER Analyst 2.0: analysis and visualization of channels and tunnels in protein structures and molecular dynamics trajectories. *Bioinformatics.* **34**, 3586–3588 (2018).
